# An oxalate cathode for lithium ion batteries with combined cationic and polyanionic redox

**DOI:** 10.1038/s41467-019-11077-0

**Published:** 2019-08-02

**Authors:** Wenjiao Yao, A. Robert Armstrong, Xiaolong Zhou, Moulay-Tahar Sougrati, Pinit Kidkhunthod, Sarayut Tunmee, Chenghua Sun, Suchinda Sattayaporn, Philip Lightfoot, Bifa Ji, Chunlei Jiang, Nanzhong Wu, Yongbing Tang, Hui-Ming Cheng

**Affiliations:** 1 Functional Thin Films Research Center, Shenzhen Institutes of Advanced Technology, Chinese Academy of Sciences, Shenzhen, 518055 China; 2 School of Chemistry, University of St Andrews, St Andrews, Fife KY16 9ST UK; 3 ALISTORE-ERI, 80039 Amiens Cedex, France; 4 Université de Montpellier 2 Place Eugène Bataillon - CC 1502, 34095 Montpellier CEDEX 5, France; 5 Synchrotron Light Research Institute, 111 University Avenue, Muang District, Nakhon Ratchasima, 30000 Thailand; 6 Department of Chemistry and Biotechnology, Center for Translational Atomaterials, Faculty of Science, Engineering and Technology, Swinburne University of Technology, Hawthorn, VIC 3122 Australia; 7 Tsinghua-Berkeley Shenzhen Institute, Tsinghua University, Shenzhen, 518055 China

**Keywords:** Batteries, Batteries

## Abstract

The growing demand for advanced lithium-ion batteries calls for the continued development of high-performance positive electrode materials. Polyoxyanion compounds are receiving considerable interest as alternative cathodes to conventional oxides due to their advantages in cost, safety and environmental friendliness. However, polyanionic cathodes reported so far rely heavily upon transition-metal redox reactions for lithium transfer. Here we show a polyanionic insertion material, Li_2_Fe(C_2_O_4_)_2_, in which in addition to iron redox activity, the oxalate group itself also shows redox behavior enabling reversible charge/discharge and high capacity without gas evolution. The current study gives oxalate a role as a family of cathode materials and suggests a direction for the identification and design of electrode materials with polyanionic frameworks.

## Abstract (web summary)

Polyoxyanion compounds are alternative cathodes to conventional oxides, but their reliance on the transition metal redox limits the performance. Here the authors report an oxalate system which possesses additional polyanionic redox reactivity, suggesting a new direction for cathode materials design.

## Introduction

Since the commercialization of lithium-ion batteries (LIBs) in 1991, LIBs have become the dominant rechargeable energy storage devices owing to their high energy density and long lifetime^1,2^. The most studied cathode materials, such as layered LiCoO_2_ and LiNi_1/3_Mn_1/3_Co_1/3_O_2_ (NMC111), are mainly based on cobalt redox processes. However, the limited availability, uneven distribution and toxicity of cobalt have made it desirable to explore new-generation cathodes for LIBs^3,4^. In this respect, iron-based polyanionic compounds are attractive cathode materials for large-scale energy storage applications since using naturally abundant iron as a redox center will effectively alleviate the restrictions of limited resources and decrease the energy cost^5–8^. More importantly, oxygen atoms are stabilized in the polyanions (e.g., (PO_4_)^3−^) via the strong covalent bonds, which could significantly reduce the risk of oxygen evolution and increase the cycling stability^9^. The successful use of lithium iron phosphate (LiFePO_4_) as a cathode for LIBs^5^ due to its low cost, high safety and long cycling life extensively stimulated the investigations of a range of polyanionic compounds, such as phosphate (PO_4_)^3−^, sulfate (SO_4_)^2−^, borate (BO_3_)^3−^ and silicate (SiO_4_)^4− ^^10–13^. Despite the comparable polarizability of the oxalate group (C_2_O_4_)^2−^ to (PO_4_)^3−^ and (SO_4_)^2−^, which affords a commensurate redox potential, oxalates have received little attention as potential cathode materials.

Recently, Tarascon et al. reported the electrochemical performance of Fe_2_(C_2_O_4_)_3_·4H_2_O as a positive electrode in LIBs^14^. This was followed by a number of studies on other oxalate cathodes^15–17^, which demonstrated that the specific capacity of these oxalates originates solely from the Fe^3+^/Fe^2+^ redox couple, indicating a promising class of polyanionic positive electrode materials for their high redox potentials. The recent discovery of anionic redox has attracted much attention because the capacity will be dramatically improved if both anionic and cationic redox reactions take place in the same cathode^18–20^. This phenomenon is found in Li-rich layered oxides, such as Li_1.2_Ni_0.13_Mn_0.54_Co_0.13_O_2_ and Li_1.2_Ni_0.2_Mn_0.6_O_2_^21,22^, but has been observed in polyanionic compounds (Supplementary Table 1). Moreover, it remains a challenge to achieve good cycling performance due to oxygen evolution at the end of the first charge and voltage fade on extended cycling^23^. The only successful attempts reported so far have to rely on platinum-group elements such as Ir and Ru which overcomes gas liberation^24,25^. Thus, developing new polyanionic compounds utilizing anionic and cationic redox couples is a promising strategy to meet the requirements in both energy density and safety.

In this work, we report an iron-based polyanionic compound in the oxalate family, Li_2_Fe(C_2_O_4_)_2_. Importantly, oxalate and iron redox couples combine during charge/discharge, while no gas generation could be detected by in-situ mass spectroscopy. This work demonstrates a dual-redox strategy in oxalate family to develop high-capacity polyanionic cathodes together with the merits of low cost, good safety and being environmentally benign.

## Results

### Structural and thermal Characterization

**Fig. 1:**
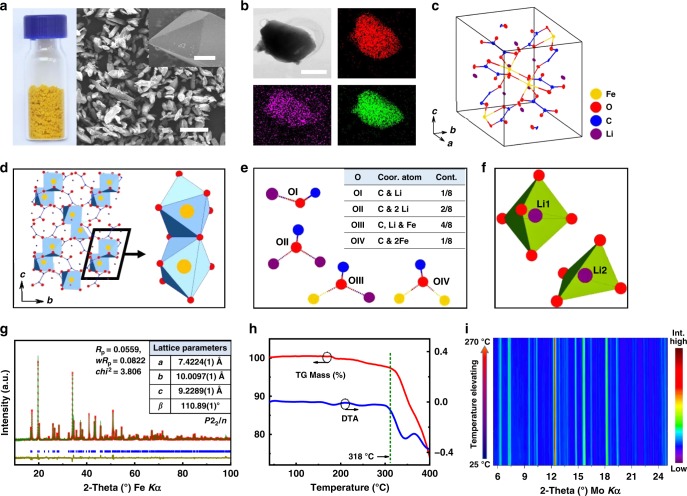
Characterization of Li_2_Fe(C_2_O_4_)_2_ (LFOx for short). **a** Photo and SEM image of as-synthesized crystallites (scale bar = 50 μm). The inset (scale bar = 5 μm)shows a polyprismatic single crystal at the size of ~30 × 20 × 25 μm. **b** TEM and corresponding EDS mapping of Fe, C, O (scale bar = 200 nm). **c** Thermal ellipsoids unit cell determined from single crystal XRD. **d** Extended structure of LFOx to show its three-dimensional framework and FeO_6_ motifs. **e** Classification of oxygen according to their nearest neighbour coordination environment. **f** Schematic lithium environments in LFOx. **g** Rietveld refinement of room-temperature powder XRD Fe *Kα*_1_ on a pristine powder sample. The inset displays the refined unit cell information. **h** TG and derived DTA curves of powder sample upon heating in N_2_ flowing gas. **i** Variable-temperature XRD from 25 to 270 °C in N_2_ gas flow (Mo K*α*)

Yellow crystals of Li_2_Fe(C_2_O_4_)_2_ (LFOx) (Fig. 1a) were synthesized via a hydrothermal process (see Methods). Scanning electron microscope images showed that these crystals have sizes of 20–50 μm and exhibit a typical polyhedral morphology. Energy dispersive spectroscopy revealed a uniform distribution of Fe, C, and O in the obtained crystallites (Fig. 1b). We used low temperature (173 K) single crystal X-ray diffraction (XRD) to determine the precise crystal structure of LFOx. The detailed crystallographic data has been deposited in the Cambridge Crystallographic Data Centre (CCDC No. 1416422). Figure 1c displays a unit cell with ellipsoidal thermal displacement. LFOx crystallizes in the monoclinic system, space group *P*2_1_/*n*, with *a* = 7.364(3) Å, *b* = 9.983(3) Å, *c* = 9.173(4) Å, *β* = 110.93(1) °, *Z* = 4. In each asymmetric unit, there are two, one, four, and eight independent sites for Li, Fe, C, and O, respectively. The atomic sites and anisotropic thermal displacements are listed in Supplementary Tables 2–3.

An extended structure viewed along the *a*-axis (Fig. 1d) reveals that each Fe atom is coordinated by six O atoms to form a distorted FeO_6_ octahedron. Interestingly, pairs of adjacent FeO_6_ octahedra share edges and form a [Fe_2_O_10_] dimer (Fig. 1d left). Meanwhile, the oxalate groups maintain almost planar motifs. In addition, [Fe_2_O_10_] dimers are connected by planar oxalate groups to form a 3D framework formulated by [Fe(C_2_O_4_)_2_]^2−^, while Li^+^ ions are located at the interstitial sites. It can be noted that the oxygen atoms in the structure can be classified into four categories according to their surrounding environment. As depicted in Fig. 1e, out of eight independent O sites in the unit cell, OI is surrounded by one C and one Li, OII by one C and two Li, OIII by one C, one Li and one Fe, and OIV by one C and two Fe (Supplementary Table 4). As for Li, there are two coordination environments (Fig. 1f), with one surrounded by five O atoms (Li1), and the other encircled by four O atoms (Li2). Bond-valence sum (BVS) calculations^26^ confirmed the oxidation states of Li and Fe as +1 and +2, respectively. Rietveld refinement of a powder XRD pattern yielded lattice parameters and atomic sites in accordance with single crystal structure, which confirms its high purity (Fig. 1g, “Methods” section and Supplementary Fig. 1).

One common concern of oxalate compounds is their thermal stability. Therefore, we investigated the thermal behavior of LFOx. As shown in the thermogravimetric (TG) curve in Fig. 1h, negligible weight loss was observed until 318 °C. The tiny weight change (<1%) around 180 °C in the TG curve may be attributed to dehydration of trace amounts of FeC_2_O_4_·2H_2_O impurity^27^, which was later detected by Mössbauer spectroscopy discussed below. Moreover, variable-temperature XRD patterns from room temperature up to 270 °C (Fig. 1i) showed no phase change, confirming the excellent stability of this phase in the chosen temperature range.

### Characterization of Fe^2+/3+^ redox activity

**Fig. 2:**
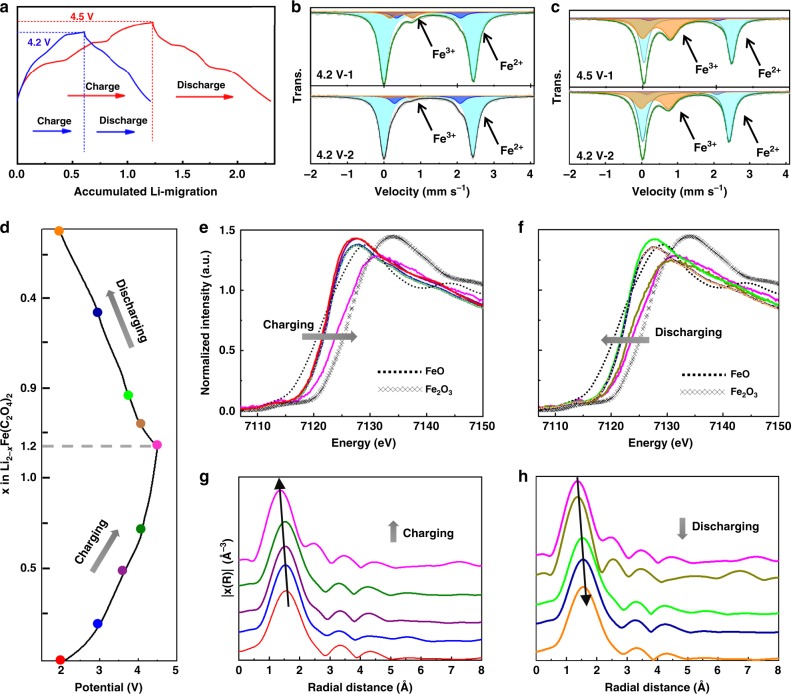
Electrochemistry and evolution of iron in the stabilized cycling process. **a** Typical galvanometric charging and discharging curves of stabilized half cells in the window of 2.0–4.2 V (blue line) and 2.0–4.5 V (red line) at a current density of 10 mA g^−1^. Mossbauer patterns of recovered electrodes after charging to 4.2 V (**b**) and 4.5 V (**c**). The up and bottom patterns in (B) and (C) correspond to different batches of samples at 4.2 V-charged and 4.5 V-charged states, respectively. The consistency of each pair verifies the repetition of the measurements. The light blue, black, and orange-filled curve represents Fe^2+^ in LFOx, Fe^2+^ in trace amount of impurity, and new Fe^3+^, respectively. **d** Load curve for in-situ cell and corresponding synchrotron Fe *K*-edge XANES during the charge (**e**) and discharge (**f**), and Fe EXAFS during the charge (**g**) and discharge (**h**). The curves in different colours in (**e**–**h**) reflect the chronological states highlighted by circles in the same colour in (**d**), namely red, blue, purple, olivine, pink, dark yellow, green, navy blue and orange. FeO and Fe_2_O_3_ were used as references for Fe *K*-edge XANES, as indicated by black dotted and grey crossed lines in (**e**, **f**). The olivine curve in (**e**) is represented by dashed line to avoid complete overlap with blue one. Same operation was applied to navy blue and orange curves in (**f**). The Fe EXAFS spectra in (**g**, **h**) were cascaded vertically to give a better clarity. The black arrows in (**g**, **h**) show the decrease and increase of Fe–O bond length during charging and discharging process

We evaluated the electrochemical properties of LFOx in coin-type half cells. Galvanostatic charge-discharge (GCD) tests were performed over different voltage windows to determine the optimum Li-extraction/reinsertion range. Typically, cells cycled between 2.0–4.2 and 2.0–4.5 V were stable after first charge (Supplementary Fig. 2), corresponding to approximate 0.625 and 1.25 Li-ion extraction/reinsertion, respectively (Fig. 2a). Cells were further tested under various current densities (50–500 mA g^−1^) in the range of 2.0–4.5 V (Supplementary Figs. 3–4). Linear sweep voltammetric (LSV) curves on a pure electrolyte cell and stabilized LFOx-based half-cell illustrated that the electrolyte and the half-cell were both relatively stable in the voltage window of 2.0–4.5 V without obvious decomposition of electrolyte (Supplementary Fig. 5). Interestingly, the obtained 1.25 Li-ion exchange in the voltage window 2.0–4.5 V clearly exceeds the expected 1.0 Li-ion extraction solely based on the Fe^2+^/Fe^3+^ redox couple. The extra capacity may originate either from the Fe^3+^/Fe^4+^ couple or anionic redox. We first employed Mössbauer spectroscopy to detect the valence states of Fe at different charge/discharge states (Fig. 2b,c and Supplementary Fig. 6 and Table 5). Although trace amounts of Fe(C_2_O_4_)·2H_2_O were detected in the pristine sample (<5%, Supplementary Table 5), this was unchanged in samples at different states of charge and discharge and therefore had negligible influence on the electrochemical process of the main phase. When charged to 4.2 V, 0.625 Li^+^ had been extracted and hence the anticipated amount of Fe^3+^ should be over 50% based on charge balance considerations, while much less than 50% Fe^3+^ was detected (Fig. 2b). When further charged to 4.5 V, 1.25 Li^+^ was extracted, meaning that all Fe^2+^ should have been oxidized to Fe^3+^ and some even to Fe^4+^ if the charge balance solely relies on Fe redox. While strikingly, ~50% of Fe remained as Fe^2+^ and the rest was Fe^3+^ (Fig. 2c), which vividly demonstrates that non-Fe redox makes important contributions to the observed capacity. The repetition of the measurements were verified by the consistency of patterns for different batches of samples at 4.2 V- and 4.5 V-charged states (up and bottom in Fig. 2b, c).

Before exploring the nature of the other redox mechanisms, iron states under different states of charge have been studied by in-situ synchrotron *X*-ray absorption near-edge spectra (in-situ XANES), aiming to reveal their electronic structures, oxidation states, and coordination symmetry. Measurements of the in-situ Fe *K*-edge were performed in transmission mode using gas ionization chambers to monitor the incident and transmitted *X*-ray intensities (see Methods). For comparison, FeO and Fe_2_O_3_ were used as references. The XANES of the pristine sample is shown in Supplementary Fig. 7. The small pre-edge peak at around 7110–7115 eV, typically representing the electric-dipole forbidden transition of a Fe 1 s electron to a Fe 3d orbital, can be attributed to a slight mixing of 3*d* and 4*p* orbitals (Supplementary Fig. 8) due to local structural distortions in the FeO_6_ octahedron^28–30^. The absence of a shoulder at ~7118 eV suggests that there were no FeO_4_ tetrahedra in our sample^30^. The XANES profile of as-prepared LFOx (pre-edge and the main edge) is quite different from the FeO, probably because FeO_6_ octahedral units in LFOx are highly distorted. However, the Fe^2+^ state can be clearly identified from the main peak of LFOx, which is very close to that of FeO. The charge/discharge curve of the stabilized in-situ cell during XANES collection exhibited a similar profile to that observed previously (Fig. 2d). Figure 2e, f show the evolution of the Fe *K*-edge during the charging/discharging process, along with quantitative analysis of the oxidation state by linear combination fitting (Supplementary Table 6). It can be seen that the shape of the *K*-edge is almost unchanged during charging/discharging (Fig. 2e, f), confirming that the FeO_6_ octahedral coordination is highly stable. The main edge shifts to higher energy upon charging, while even to 4.5 V, a clear distance between the main peak of LFOx and Fe_2_O_3_ can be observed, indicating that Fe^2+^ has been only partially oxidized to Fe^3+^, in agreement with the Mössbauer spectra. Similar analysis has been performed for the discharging process, reaching the same conclusion that the iron states are not linearly changed from Fe^2+^ to Fe^3+^. The XANES curves for samples at the beginning and the end of the cycle match well with each other, indicating the change of iron state was relatively reversible (Supplementary Fig. 9).

Extended synchrotron *X*-ray absorption fine-structure (EXAFS) was further employed to explore the bond length and local structure around the Fe atomic center during charging/discharging, and the corresponding Fourier transform (FT) spectra are shown in Fig. 2g, h and Supplementary Fig. 10. The FTs of the *k*^2^-weighted Fe *K*-edge EXAFS oscillations were calculated within *k* = 2.6–10 Å^−1^ (this range being chosen so as to minimize noise), with detailed pseudo-radial structure-function results tabulated in Supplementary Table 7. Without phase-shift correction (normally 0.2–0.3 Å shorter distances compared with the real bond distances), the first peak at about 1.5 Å represents Fe–O bonds in the FeO_6_ octahedra, while the peak at 3.1 Å corresponds to edge-sharing Fe–Fe. Upon charging, the Fe–O bond lengths shifted gradually towards lower value, indicating Fe–O bond contraction (Fig. 2g)^23,30,31^. A new peak emerged at around 2.5 Å^−1^ in the 4.5 V-charged sample, which is most likely the result of lattice distortion in the sample. The Fe–O bonds recovered gradually during discharge (Fig. 2h and Supplementary Fig. 10), indicating good reversibility of the process.

### Characterization of oxalate redox activity

**Fig. 3:**
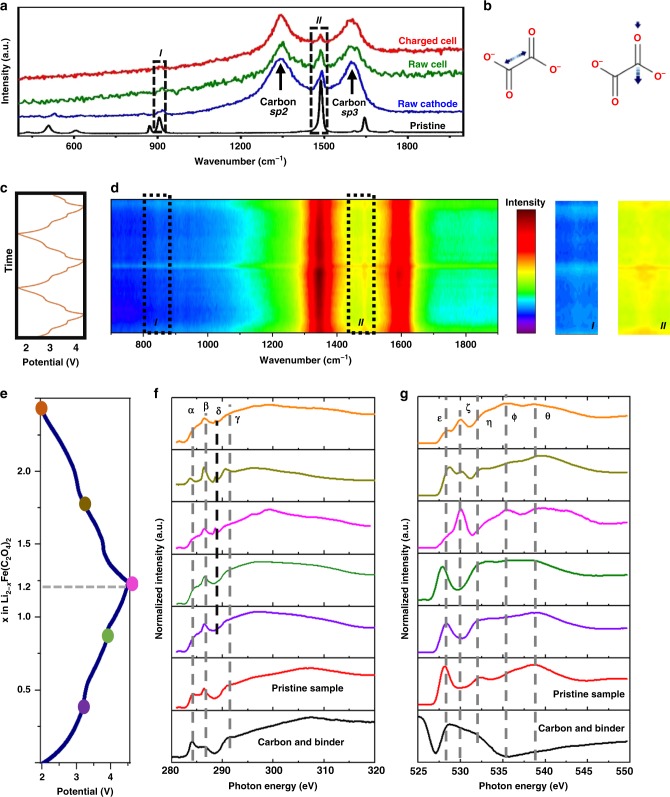
Anionic reaction mechanism investigation of LFOx. **a** Raman spectra of LFOx in states of pristine (black), raw composite cathode (blue), initial cathode in in-situ cells (green), and charged cathode in in-situ cells (red). **b** Symmetric C–C stretching and asymmetric C = O stretching corresponding to *I* and *II* peaks as framed in (**a**). **d** In-situ Raman spectra of LFOx when cycled in the window 2.0–4.5 V as displayed in (**c**). The characteristic peaks between 800–900 and 1450–1550 cm^−1^ are highlighted in the right hand column. **f**, **g** Evolution of carbon and oxygen synchrotron NEXAFS spectra corresponding to diverse states as dotted in the charge and discharge process in (**e**). All EXAFS are normalized. The bottom black lines in (**f**, **g**) are the reference composed of carbon conductor and additive in the same ratio as in composite cathode. Detailed assignments of peaks in (**f**, **g**) are stated in the main text

The above investigations confirmed that Fe undergoes the Fe^2+^/Fe^3+^ redox during Li de-/insertion in LFOx, but such cationic redox alone cannot explain the capacity obtained. Now we turn to the investigation of anionic redox couple. As gas releasing has been a common phenomenon in reported anionic redox-active cathode^21–23^, time-resolved in-situ mass spectroscopy was applied to detect if the oxalate group in LFOx cathode was decomposed to CO_2_ during charging and discharging. The results demonstrated that the cell was stable without CO_2_ generation, even holding at 4.5 V state (see “Methods” section and Supplementary Fig. 11). The geometries and electronic states of oxalate were then examined using Raman and soft X-ray Absorption Spectra. Figure 3a shows Raman spectra of the pristine LFOx (black), LFOx mixed with conductive carbon for cathode (blue), initial cathode (green) and charged cathode in in-situ cells (red). Corresponding assignments of Raman peaks of pristine LFOx are stated in Supplementary Fig. 12 and Supplementary Table 8. In Fig. 3a, the strong and broad Raman peaks at 1342 and 1600 cm^−1^ are consistent with D-band and G-band vibrations, respectively, originating mainly from conductive carbon black and additive. The characteristic peaks at *I* (917 cm^−1^) and *II* (1485 cm^−1^) arose from LFOx, which were assigned to C–C symmetric and C = O asymmetric stretching^32–34^, as schematically illustrated in Fig. 3b. Other peaks from the pristine sample become difficult to identify after assembling the in-situ cell, mainly because of noise from carbon additive, electrolyte, cell window, etc. Comparing the spectra of initial and charged cathodes in in-situ cells (green and red in Fig. 3a), it is apparent that both *I* and *II* became weaker after charging, indicating that the concentration of corresponding C–C and C = O groups is decreasing, evidence of geometry changes of the oxalate groups. Additionally, peak *II* became narrower and shifted towards higher wavenumbers in charged states (Supplementary Fig. 13), indicating that the double bond in C = O becomes more localized and stronger. The intensity evolution for the peak *I* and *II* as a function of time confirmed the periodic changes of peak *I* and *II* (Supplementary Fig. 14). Further, from in-situ Raman patterns (Fig. 3c, d), the intensity of peaks (*I* and *II*) experienced periodic fluctuations during cycling, demonstrating excellent reversibility of these changes.

Synchrotron near-edge X-ray Absorption Fine Structure (NEXAFS) spectra were further employed to detect whether the carbon (C) or oxygen (O) of the oxalate groups contribute to the redox couple, which is essential to understand the observed capacity. Different states labeled by colored circles in Fig. 3e were selected, and the NEXAFS patterns of C and O are shown in Fig. 3f, g. All NEXAFS are normalized using the intensity of the incident photon beam (*I*_0_). For comparison, carbon conductor and additive in the same ratio as in LFOx composite cathode was also measured (bottom black lines, Fig. 3f, g). The C *K*-edge spectra (Fig. 3f) display α, β, δ, ɣ signals, which are assigned to C π*, O-C = O group, C = O group, C δ*, respectively^35^. It can be seen that during the charging process (purple to green to pink), both α and γ peaks become weaker, ß peak is broadened, and new δ peak emerged. This provide evidence that the oxalate is losing its conjugated character. While during the discharging process (pink to yellow), α and γ peaks become stronger, ß peak become sharper, and δ is softened. This is opposite to the changes in the charging process, indicating an opposite reaction is happening during discharge. Similarly, in Fig. 3g, from purple to green to pink plot, η peak is broadened and the ε peak lost intensity, suggesting the oxygen bridging C and Fe is changing its electronic state. The pink plot displays clear ζ and φ peak, corresponding to O-Fe^3+^ and C = O motif, respectively. From pink to yellow, ζ and φ are weaker, while η and ε are clearer, indicating a reverse reaction^36^. The discrepancy of the orange plot in Fig. 2f, g is possibly originated from the long exposure of the measured sample in atmosphere (see Supplementary Information).

### Computational investigation

**Fig. 4:**
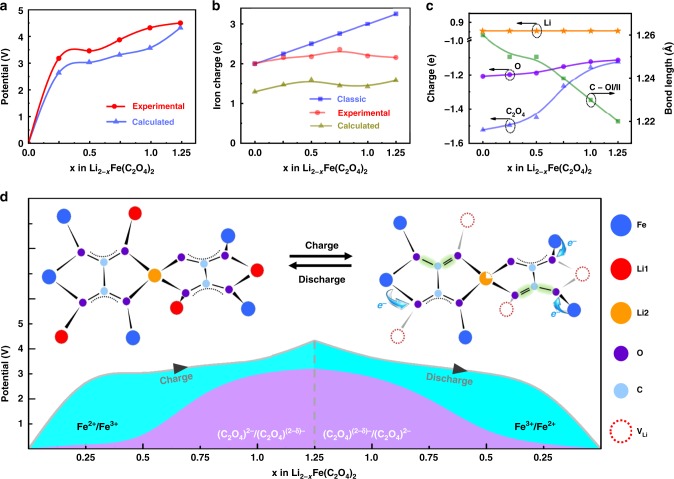
Computational investigation. **a** Potential, (**b**) iron charges, (**c**) averaged charges for Li, O and C_2_O_4_ group and calculated mean bond length of C-OI and C-OII versus Li-content in LFOx. **d** Schematic illustration of iron and oxalate redox mechanism and their contribution to specific capacity in the stabilized charging and discharging process

As demonstrated above, the accumulated experimental data point to the co-existence of cationic and anionic redox processes in LFOx. To establish a full picture of the electronic processes associated with these redox couples, first principle calculations^37^ were performed (further computational details given in Methods). To evaluate the charge and discharge performance, we start from the calculation of open-circuit voltage (OCV), as shown in Fig. 4a, which agrees well with the experimental result, confirming the validity of such calculations. It is worth to note that, the maximum capacity measured in this study is 150 mAh g^−1^, corresponding to approximately five Li-atoms removed from the ideal Li_8_Fe_4_(C_2_O_4_)_8_ unit cell, i.e., more than half can be released. Those would result in a difficulty to understand the charge balance because for LixFe_4_(C_2_O_4_)_8_, such balance has been achieved with Li and Fe donating electrons to C_2_O_4_ group, which can be described by a general equation,

1 $${\mathrm{4\delta }}\left( {{\mathrm{Fe}}} \right){{ \, + \, x\delta }}\left( {{\mathrm{Li}}} \right) = {\mathrm{8\delta }}\left( {{\mathrm{C}}_{\mathrm{2}}{\mathrm{O}}_{\mathrm{4}}} \right),$$

where δ(Fe), δ(Li), δ(C_2_O_4_) are the charges for Fe, Li and C_2_O_4_, respectively. Based on the classical valence-bond theory (VBT), it leads to δ(Fe) = +2, δ(Li) = +1, and δ(C_2_O_4_) = −2 when Li is fully incorporated with *x* = 8. However, such balance has to be broken with Li-removal. For instance, when *x* < 8, δ(Fe) has to be linearly increased to maintain charge balance if still following VBT with constant δ(Li) and δ(C_2_O_4_), as shown in Fig. 4b (blue line); as a result, Fe will reach fully oxidized state (+3) with *x* = 4, giving a theoretical capacity of ~110 mAh g^−1^, which is lower than the observed capacity (150 mAh g^−1^). In addition, the measured δ(Fe), as shown in Fig. 4b (red line), deviates notably from the linear relationship, indicating that the electron loss due to Li-extraction cannot be fully compensated from iron. Under this context, Bader charges^38^ have been calculated to clarify how the charge balance has been established. Calculated δ(Fe) is presented in Fig. 4b (yellow line), which clearly reproduces the observed fluctuation of δ(Fe) (Fig. 2) although the Bader charge is smaller than the measured values due to the localization and delocalization consideration in charge analysis. Therefore, the assumption that δ(Li) and δ(C_2_O_4_) keep constant is not well satisfied, meaning that sole cationic redox cannot support the observed capacity (150 mAh g^−1^).

From the calculated Bader charges, δ(Li) is almost a constant with the value of 0.87, but δ(C_2_O_4_) varies remarkably, as shown in Fig. 4c, which provides direct evidence of polyanionic redox in this process. More importantly, the C–O bond length varies simultaneously with δ(C_2_O_4_), which matches well with our in-situ Raman and synchrotron NEXAFS measurements. As demonstrated in Eq.^1^, the anionic redox exactly helps the charge balance and can play a key role to achieve higher capacity than that dominated by cationic redox, as shown in Fig. 4d, in which the capacity associated with anionic and cationic redox are separated and highlighted by blue and purple colors. Impressively, the oxalate contribution starts from the beginning, and dominates after 75 mAh g^−1^ has been extracted. This is a vivid demonstration that both anionic and cationic redox are important, and the co-existence can reach the observed capacity (150 mAh g^−1^) without Fe^3+^/Fe^4+^ couples. The merit can be established from a ‘if-not-so’ strategy: if the capacity is solely contributed from cationic redox, most Fe-atoms have to be oxidized to Fe^3+^ and partially to Fe^4+^; as a result, FeO_6_ octahedra has to be partially turned to new Fe–O coordination to achieve such high oxidation. Potentially, O-redistribution results in the destabilization of anionic groups because more oxygen needs to be provided to stabilize highly oxidized iron. Under the dual-redox scheme, however, the loss of donated electrons associated with Li extraction can be balanced by increased donation from Fe (cationic redox, highlighted by the arrow in Fig. 4d) and decreased demand from oxalate (anionic redox). Specifically, anionic redox can be achieved through changing the resonance of C–O bonding (dotted line) to slightly localized C = O, which avoids excessive oxidation of Fe and heavy structural distortions. Therefore, the co-existence of cationic and anionic redox not only contributes to the capacity, but also helps to achieve excellent electrochemical durability, as demonstrated above.

## Discussion

Transition metal oxalates provide numerous chemical and structural options to explore positive electrode materials. In this work, a dual-redox polyanionic cathode is presented in the oxalate family. The robust 3D structure of LFOx provides highly reversible Li de-/insertion while no CO_2_ could be detected in the in-situ MS measurements. Collaborative interaction between iron and oxalate appears to be the fundamental factor that modulates polyanionic and cationic redox couples. Although the electrochemical performance of this oxalate may be further improved by reducing its size, modifying the interfacial structure and optimizing the electrolyte systems, etc, the results presented here clearly indicate the feasibility of the realization of harmonic and comparable cationic and anionic redox in one polyanionic compound. This dual redox strategy offers a fresh option to develop high-performance cathode materials with merits of high capacity, good stability, low cost as well as being environmentally friendly.

## Methods

### Materials

Iron(II) chloride tetrahydrate (FeCl_2_·4H_2_O, ≥99%), oxalic acid dihydrate (H_2_C_2_O_4_·2H_2_O, ≥99%), and lithium carbonate (Li_2_CO_3_, ≥99%) were purchased from Sigma-Aldrich. Li foil (thickness of ~100 μm), poly(tetrafluoroethylene) (PTFE, 1 μm powder size), conductive carbon black, propylene carbonate (PC), ethyl carbonate (EC), dimethyl carbonate (DMC) were purchased from Sigma-Aldrich. Kynar Flex 2801 (a copolymer based on polyvinylidene fluoride PVDF) binder was purchased from Arkema Group. The glass fiber separator (Whatman, 47 mm) was purchased from Shanghai Huanao Technology Ltd. All chemicals were used directly as received without further processing.

### Synthesis and structure of Li_2_Fe(C_2_O_4_)_2_ crystallites

Single crystals of Li_2_Fe(C_2_O_4_)_2_ were prepared by hydrothermal method at the mild condition. FeCl_2_·2H_2_O, H_2_C_2_O_4_·2H_2_O, and Li_2_CO_3_ were mixed in a Teflon-lined autoclave in the molar ratio of 1.5:4:3 (1 for 5 mmol). The autoclave was sealed and kept at 190 °C for 7d and then cooled down in the air. The resulting products were repeatedly washed with distilled water and acetone to remove Li_2_C_2_O_4_ and Li_2_CO_3_ by-product, and then dried at 60 °C overnight. Single crystal XRD data were collected on a Rigaku SCX Mini diffractometer using Mo *K*α radiation (*λ* = 0.710 73 Å) at 173 K. Rigaku CrystalClear 2.0 was employed to index and process the raw data. The structure was then solved by direct methods and refined using SHELX-2014 incorporated in the WinGX program^39^. All atoms were refined anisotropically. Absorption corrections were performed semi-empirically from equivalent reflections on the basis of multi-scans.

### Basic characterization

Scanning electron microscope (SEM) measurements were carried out on Hitachi S-4800 equipped with an energy dispersive X-ray (EDX) detector. Powder XRD patterns were recorded on a Stoe STADI/P diffractometer operating in transmission mode with Fe *K*α1 radiation (*λ* = 1.936 Å) in the 2θ range 10–100°, the total data collection time being 16 h. Data sets were refined by conventional Rietveld methods using the GSAS package with the EXPGUI interface^40^. The background, scale factor, zero point, lattice parameters and coefficients for the peak shape function were refined until convergence. For variant temperature XRD, hand-ground samples were sealed into flowing N_2_ when patterns being recorded on a PANalytical Empyrean diffractometer equipped with an Anton Paar HTK1200N furnace. The diffractometer was operating in reflection mode with Mo *K*α radiation (*λ* = 0.7093 Å) in the 2*θ* range 5.5–24.5° when heating from room temperature to 270 °C at various intervals. Thermogravimetric analysis (TGA) was carried out with ground crystals of LFOx using a NETZSCH TG 209 thermal analyser. A sample (about 10 mg) of ground crystallites was placed in an alumina crucible and heated from room temperature to 400 °C at a rate of 5 °C min^−1^ in flowing N_2_ atmosphere. The mid-infrared (IR) spectrum was obtained at room temperature using a Perkin Elmer Spectrum GX IR spectrometer. The spectra were collected in the range 400 to 4000 cm^−1^ with resolution of 1 cm^−1^. Raman spectra (HORIBA, XploRA PLUS) were obtained in the backscattering mode using an Ar^+^ laser with a wavelength of 514.5 nm at a power of 5 mW. The probe aperture was near 10 μm, the wavelength resolution was 1 cm^–1^ in the range of 500–2000 cm^−1^.

### Electrochemical characterization

Samples from hydrothermal method were first ball-milled for 30 min using a Fritsch Pulverisette 8 mill with 30% w/w Kejten black carbon to improve the conductivity of LFOx. Samples before and after ball-milling were both examined with XRD, IR, Raman and SEM-EDX to ensure the effective nanonization and even dispersion of ingredients (Supplementary Figs. 15–19). The subsequent powder was ground with binder (polytetra-fluoroethylene, PTFE, 10%) until homogeneous. Cathode pellets (PTFE as binder) with 6–10 mg cm^−2^ active material were tested in coin cells (CR2325, NRC Canada) with Li metal as anode, LP30 (1 M LiPF_6_ in EC: DMC = 1:1) as electrolyte. The coin cells were installed in an argon-filled glove box with both water and oxygen contents less than 0.1 ppm. Half cells were then tested by galvanostatic cycling in various current from 50 mA g^−1^ to 500 mA g^−1^ in the voltage window of 2.0–4.2 V, 2.0–4.5 V or 2.0–4.6 V using a Maccor system. Gravimetric capacities are calculated based on the weight of cathode material. Cyclic voltammogram were recorded on a half cell at the scan rate of 0.2 mV s^−1^ in the window of 2.0–4.5 V. Linear sweep voltammetry measurements of pure electrolyte cells and stabilized LFOx half cells were performed. The cell used in in-situ Raman measurement was design and made by authors according to Raman equipment and cell test condition requirement, which called self-designed cell in this paper, shown in Supplementary Fig. 18. In-situ mass spectroscopy (MS) measurements were recorded on an in-situ half-cell by Hiden Analytical HPR-20 R&D. The details are stated in Supplementary Information.

### Mössbauer spectroscopy

Room temperature Mössbauer spectra were recorded on absorbers prepared under argon (coffee-bags). Each absorber contains 30–40 mg cm^−2^ active material recovered by washing with dimethyl carbonate (DMC) in an argon-filled glove box. The spectrometer is operating in the constant acceleration transmission geometry. The *γ*-ray source (^57^Co/Rd, 850 MBq) is maintained at room temperature. The isomer shift scale is calibrated using pure *α*-Fe standard. The obtained data are fitted using least-squares method and a combination of Lorentzian lines with MOSFIT program^41^. In the studied samples, each iron environment is fitted with a doublet with four characteristic parameters, detailed in Supplementary Information.

### Synchrotron X-ray measurements

Tests were carried out at Synchrotron Light Research Institute (SLRI, public organization), Thailand^42,43^. The beamline photon source covers an energy range of 40–1040 eV at the resolving power of 10,000. The synchrotron radiation source at the storage ring was generated using a beam energy of 1.2 GeV. In-situ synchrotron-based X-ray absorption spectroscopy: X-ray absorption near-edge spectra (XANES) and extended X-ray absorption fine-structure (EXAFS) of Fe *K-*edge data of the samples were collected at the SUT-NANOTEC-SLRI XAS beamline (BL5.2). The in-situ coin cells were installed using CR2016 case with two-side kapton windows to allow X-ray transmission (Supplementary Fig. 20). Typical loading of LFOx is 3–5 mg cm^−2^. A detailed investigation of the fine structure at the adsorption edges of light elements on *ex-situ* samples, i.e., carbon (C) and oxygen (O) were measured by near-edge X-ray absorption fine structure (NEXAFS) technique using total electron yield (TEY) mode at the Beamline 3.2Ua&b. The light polarization was parallel to the surface at any incident light angle. The intensity of the incident photon beam (*I*_0_) was monitored at a gold mesh in front of the samples, enabling the TEY signal to be normalized by *I*_0_. The total energy resolution was approximately 0.5 eV. Details in Supplementary Information.

### Calculations

Spin-polarized calculations under the scheme of density functional theory^37^ have been carried out for geometry optimizations, total energies, and electronic structures. In these calculations, revised Perdew-Burke-Ernzerhof functional^44^ and plane waves with a cut-off energy of 380 eV have been employed, together with the use of ultrasoft pseudopotentials for all elements except hydrogen. The details are stated in Supplementary Information.

## Supplementary information

Supplementary Information

## Data Availability

The data supporting the findings of this study are available from the authors on reasonable request. The research data supporting this publication can be accessed at 10.17630/bf533b6a-bb98-4131-9625-8e6570f1cb42.
