# Supplementary material for: An oxalate cathode for lithium ion batteries with combined cationic and polyanionic redox
Source: Nat Commun. 2019 Aug 2;10:3483. doi: 10.1038/s41467-019-11077-0 (PMC6677734; doi:10.1038/s41467-019-11077-0)
Supplement: Supplementary file 1 — Supplementary Information [file 41467_2019_11077_MOESM1_ESM.pdf]

Supplementary Information for

An oxalate cathode for lithium ion batteries with combined cationic and polyanionic redox

Yao et al.

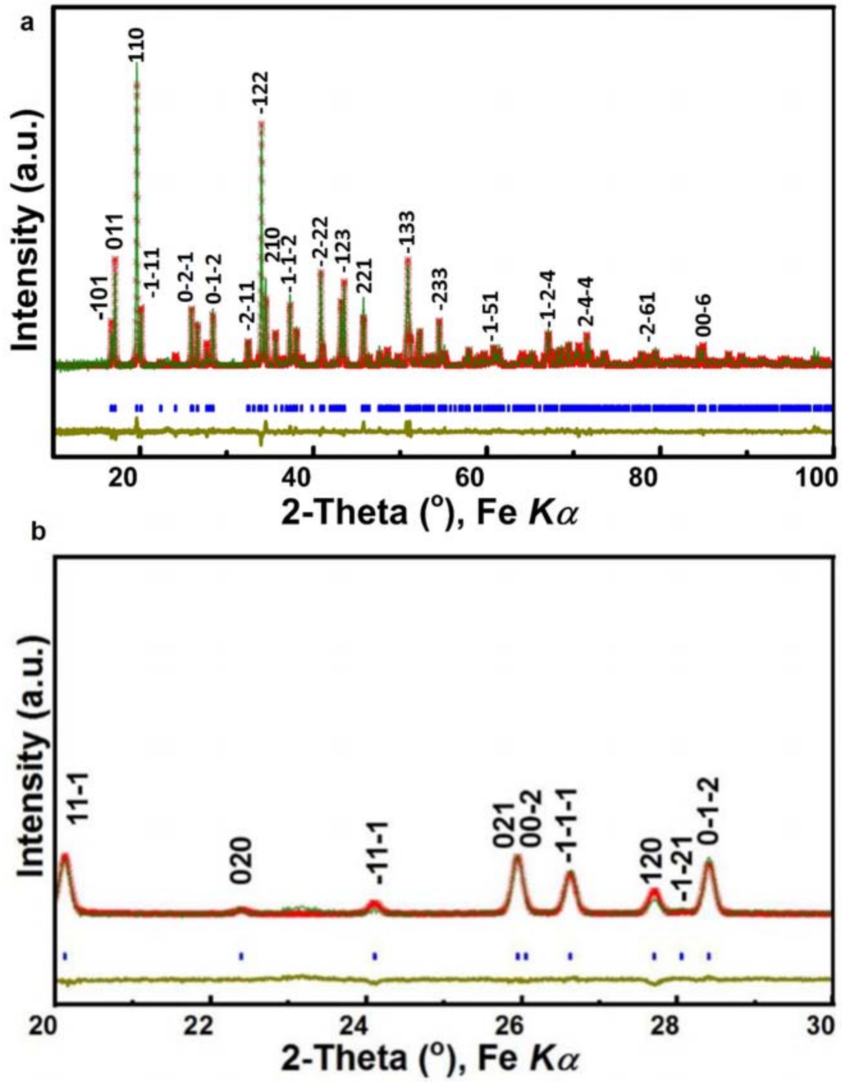

Supplementary Figure 1. Rietveld refinement of room temperature powder XRD (Fe *Kα*) on LFOx. a, Fitting pattern in the range of 10-100°. **b**, Detailed fitting pattern in the range of 20-30°. $P2_1/n$ space group, a =7.4224(1) Å, b = 10.0097(1) Å, c = 9.2289(1) Å, β = 110.89 (1)°. R_p = 0.0559, wR_p = 0.0822, χ^2 = 3.806. The green, red, blue and dark yellow in the figure stands for calculated, experimental, theoretical, and difference between calculated and experimental, respectively. The cell parameters are a little larger than those of single crystal XRD, due to thermal expansion.

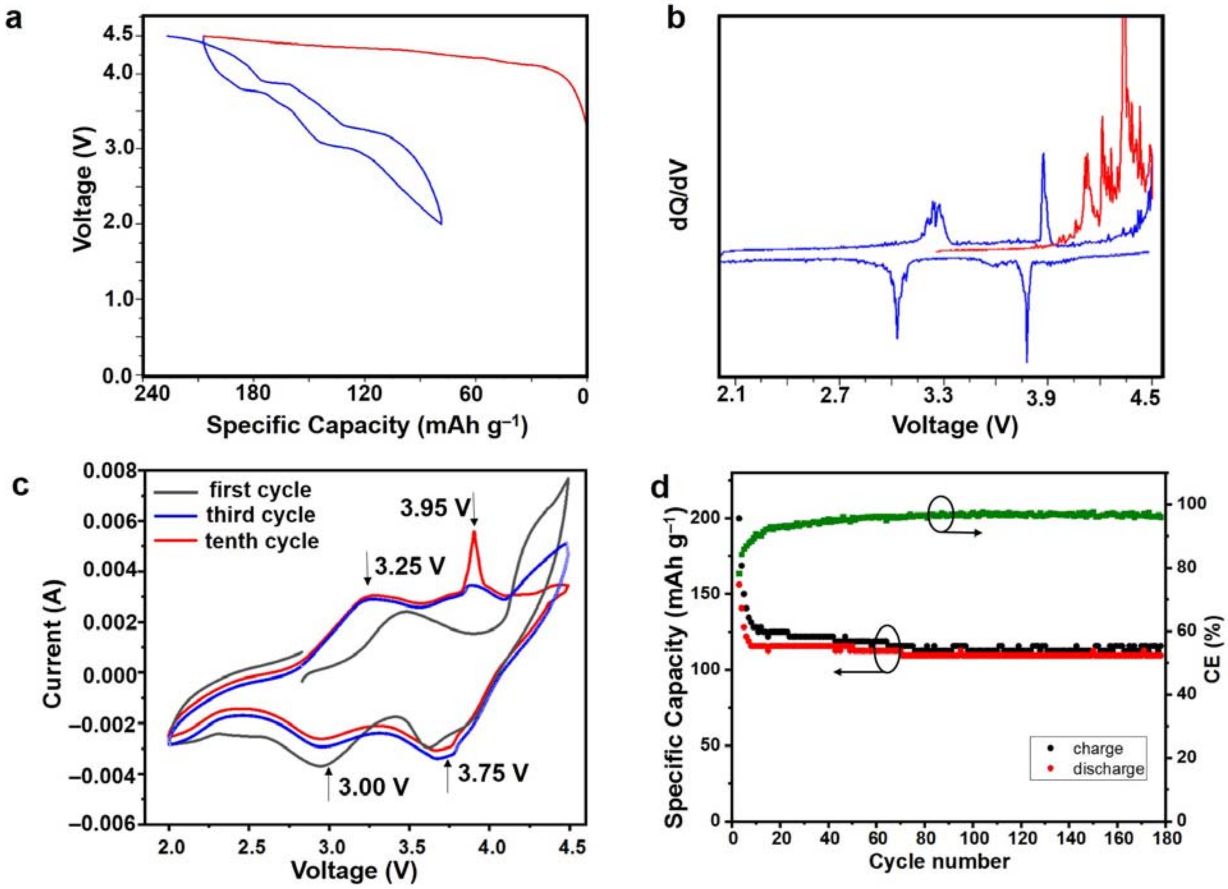

Supplementary Figure 2. Electrochemical characterization. a, Galvanostatic charge-discharge curves at the current density of 20 mA g^{-1}, and (**b**) deduced dQ/dV curve. **c**, Cyclic voltammogram curve of a stabilized half-cell, specifically, the tenth cycle, at the scan rate of 0.2 mV s^{-1} in the window of 2.0-4.5 V. Two distinctive plateaus can be clearly detected from the stabilized cell. The first charging process in **a**-**b** may contain the formation of the passivation layer on the electrodes, i.e., solid-electrolyte interphase and cathode electrode interphase (CEI), which also lead to the irregular dQ/dV curve of the first charging process. **d**, Cyclic performance under the current density of 50 mA g^{-1}.

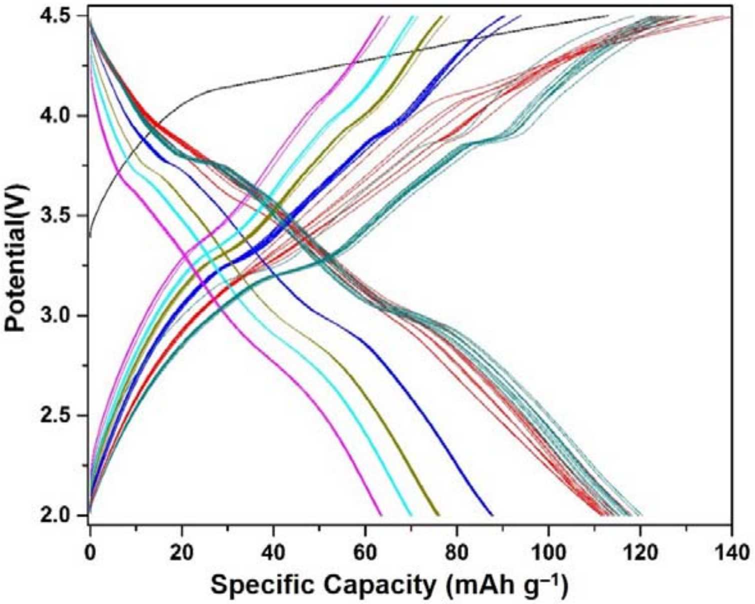

Supplementary Figure 3. Rate capability measurements. Profiles of rock-chair charging/discharging of a half-cell using 1 M $LiPF_6$ in EC and DMC (v:v=1:1) as electrolyte, under the current density of 50 (red), 100 (blue), 200 (dark yellow), 300 (turquoise), and 500 (magenta) mA g^{-1}, respectively. The black curve is the first charging-discharging process, similar to Supplementary Figure 2, while the charge capacity is much lower. This is possibly because the side reactions to form the passivation layer on the electrodes is kinetics sluggish and therefore insufficient at the larger current density. It also caused a continuous side reactions at high voltage during the next several cycles, and corresponding Couloubic efficiencies are relatively low. The teal curves represent the slow charging/discharging process under the current density of 50 mA g^{-1} after previous high rate process. Ten cycles were conducted for each current. It is shown that the profiles are highly overlapped at each current density, and the cell still delivered a comparable capacity after fast charge and discharge.

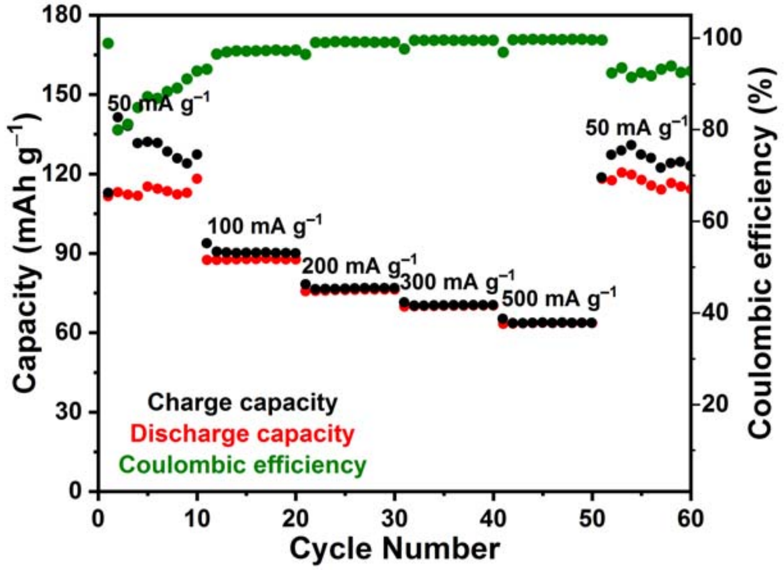

Supplementary Figure 4. Cycling performance in rate capability test. Specific capacities and corresponding Coulombic efficiency (CE) of LIBs using 1 M $LiPF_6$ in EC+DMC (1:1) as electrolyte. Ten cycles were conducted for each current. The relatively low CE comes possibly from continuous side reactions to form the passivation layer on the electrodes.

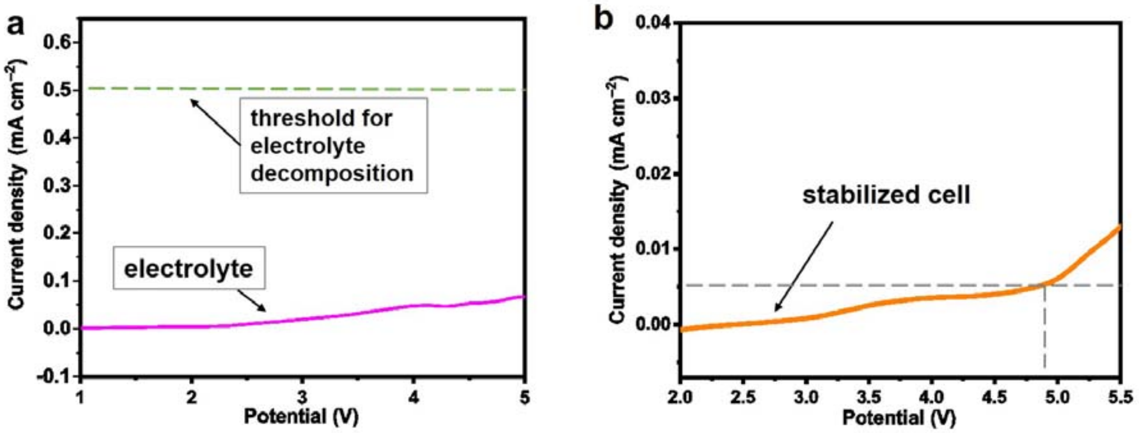

Supplementary Figure 5. Linear sweep voltammetry (LSV) measurements. a, Pure electrolyte. **b**, A stabilized LFOx half-cell. It is shown that the oxidative current densities of pure electrolyte cell fall far below the cutoff current density of 0.5 mA cm^{-2} in the pure electrolyte cell, which was used as a standard potential to identify if serious electrolyte decomposition took place, thus demonstrating the steadiness of the electrolyte. Meanwhile, the LSV curve for stabilized half-cell, is relatively flat before 4.5 V, and gradually goes up after 4.7 V, illustrating that the side reaction would become serious when voltage is higher than 4.7 V.

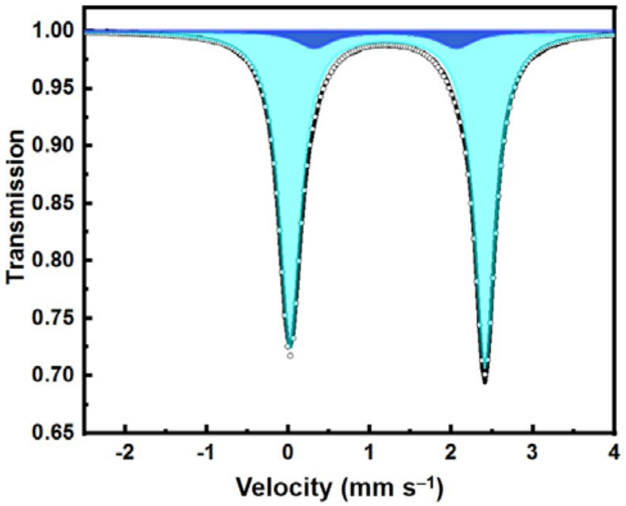

Supplementary Figure 6. Room temperature Mössbauer spectrum of pristine LFOx. The cyan and blue curves indicate original iron sites in LFOx, and original Fe site in $Fe(C_2O_4) \cdot 2H_2O$ impurity, respectively. Noteworthy the impurity (blue) is constant with that in charged states (Fig. 2 **b**,**c**), indicating that it had no influence on the studied main phase. The quantitative analysis is stated in Supplementary Table 5.

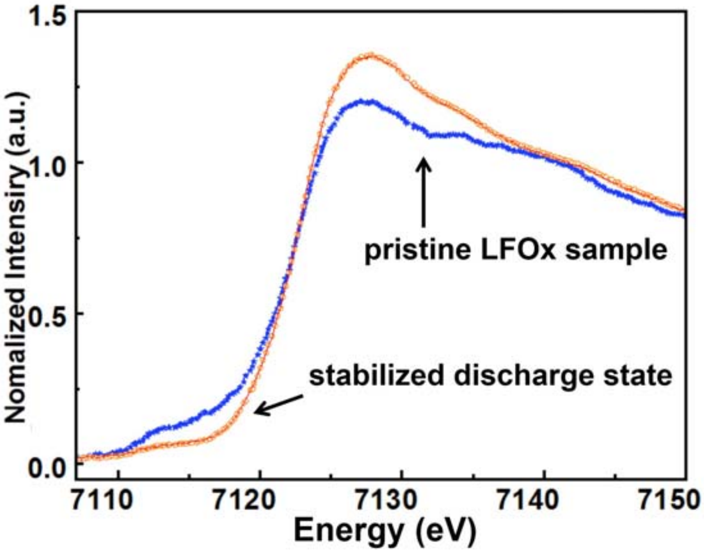

Supplementary Figure 7. Fe *K*-edge XANES spectrum of pristine $Li_2Fe(C_2O_4)_2$ sample (blue) in comparison with those of stabilized 2.0 V- state (orange and red).

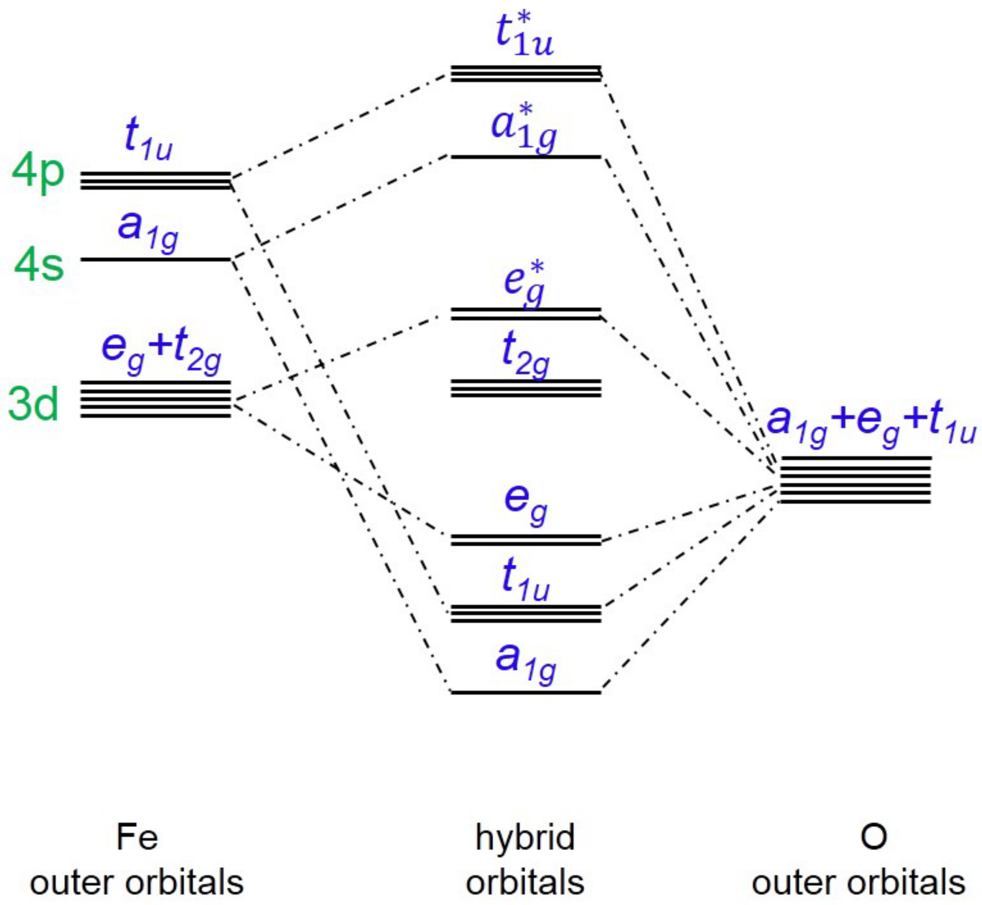

Supplementary Figure 8. Schematic diagram of FeO6 hybrid orbitals in $Li_2Fe(C_2O_4)_2$. It is shown that when the Fe is coordinated by O atoms, the 3d, 4s and 4p orbitals of Fe are rearranged to form hybrid orbitals.

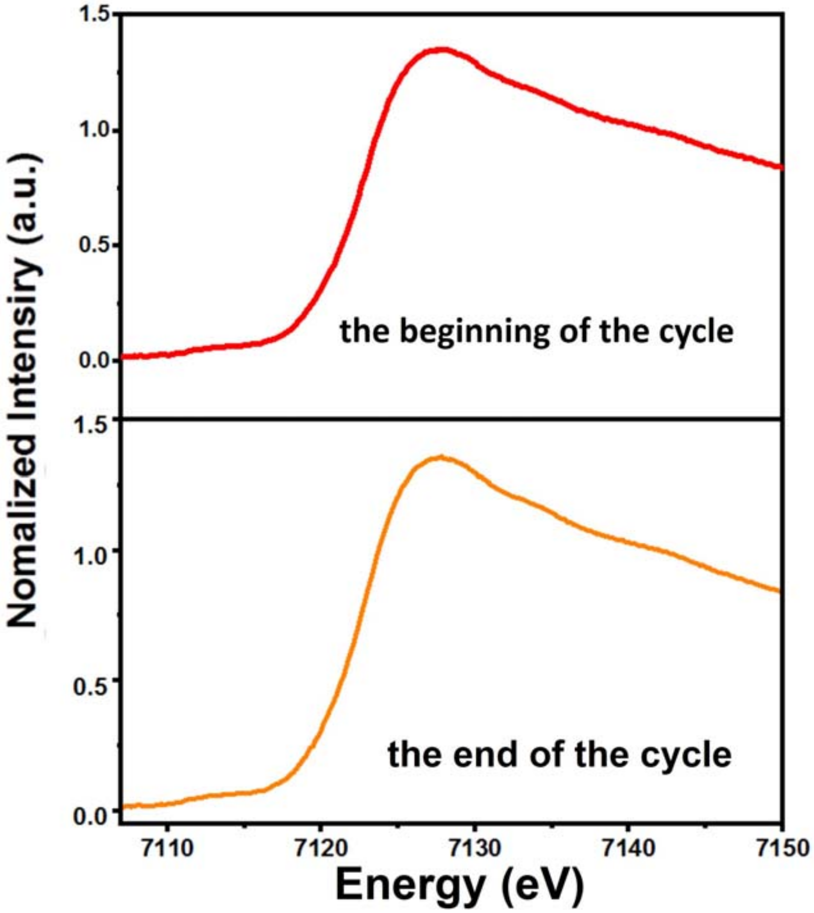

Supplementary Figure 9. Detailed comparison of the Fe *K*-edge XANES curves for samples at the beginning and the end of one cycle in the *in-situ* synchrotron XAS measurements.

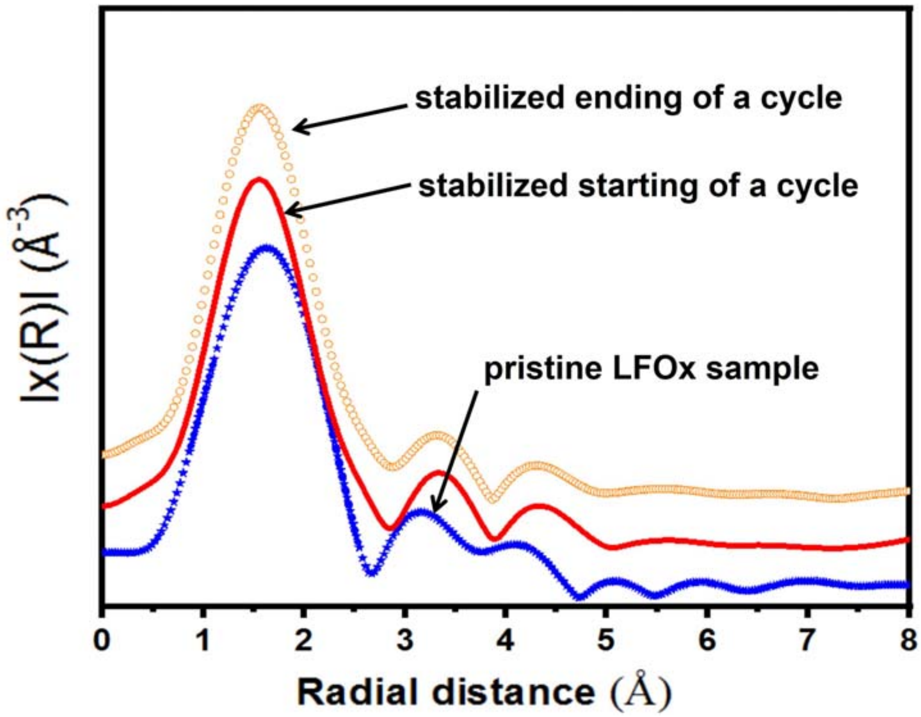

Supplementary Figure 10. Comparison of the EXAFS patterns for the samples at the starting point of charging process (orange), the end of discharging point (red), and pristine sample (blue).

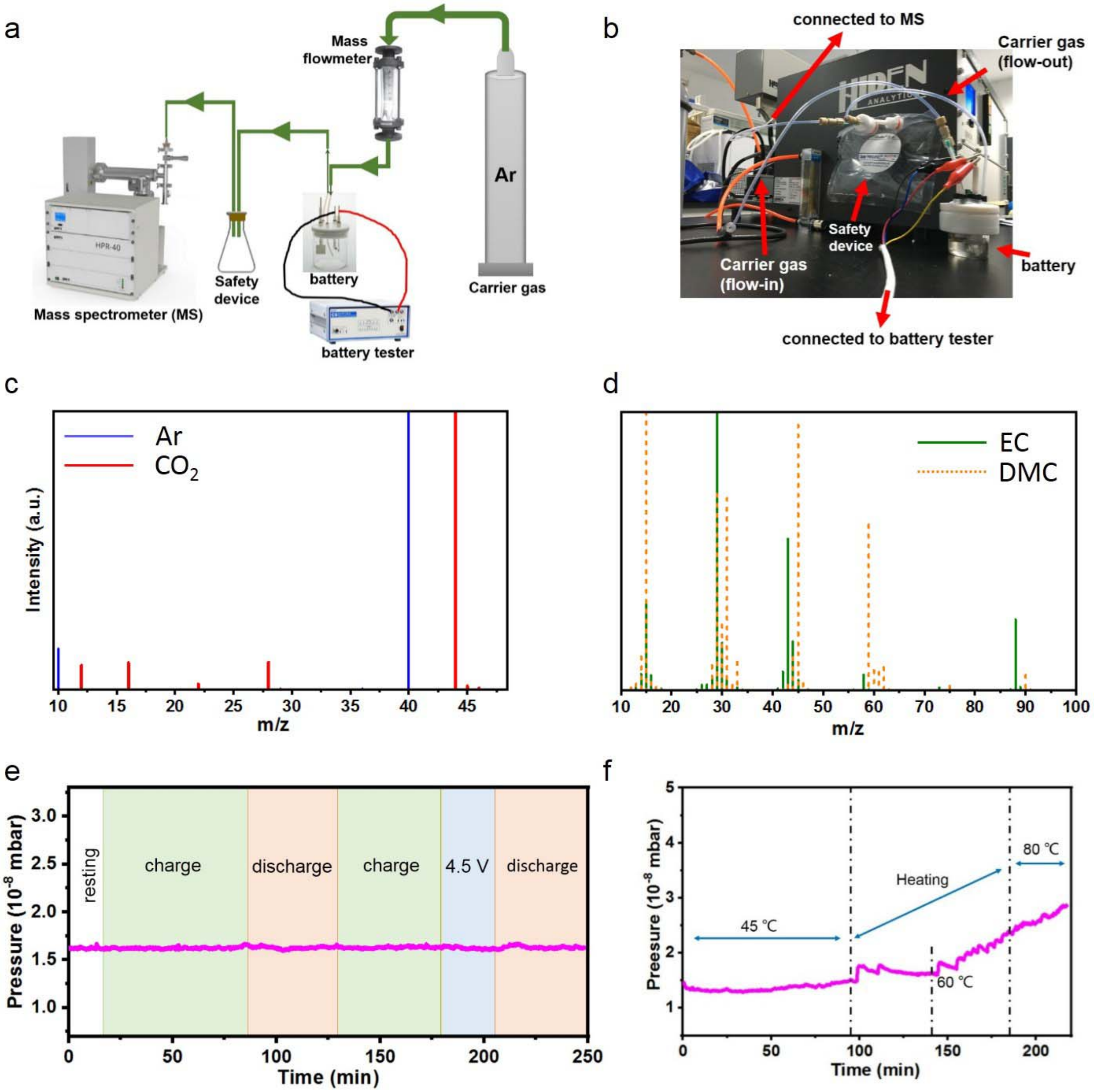

Supplementary Figure 11. *In-situ* mass spectra (MS) on LFOx-based half-cell. **a**, The schematic diagram of self-built in-situ MS system. **b**, A photo of in-situ MS testing system. **c**, The standard MS patterns of Ar (blue) and CO_2 (red). **d**, The standard MS patterns of EC (green) and DMC (yellow). **e**, Time-resolved in-situ MS pattern on an in-situ battery which were left resting, cycling, charging to 4.5 V and holding at this voltage, and at last discharging to 2.0 V. **f**, Variant temperature *in-situ* MS pattern for a 4.5 V-charged cell from 45°C to 80°C.

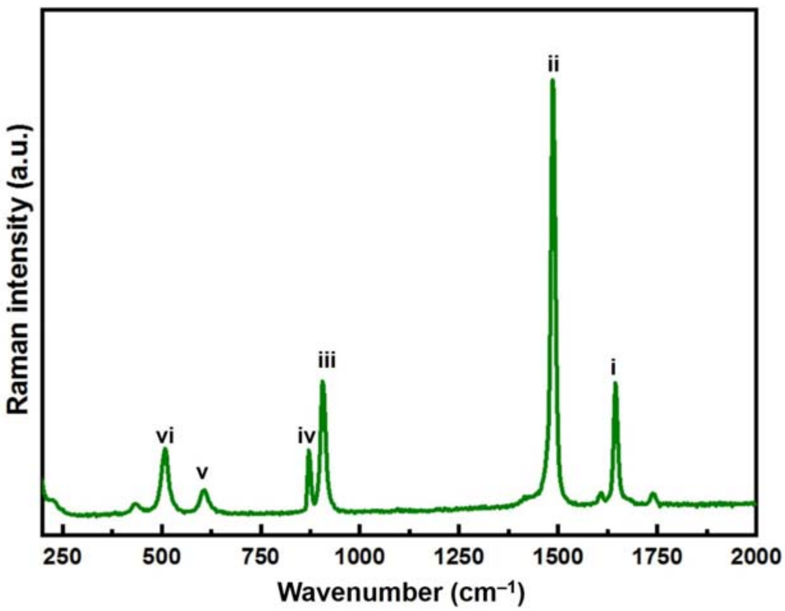

Supplementary Figure 12. Raman spectrum of pure LFOx crystallites from 200 −2000 cm^{-1}. This region is chosen since the IR test has demonstrated no OH group in the samples. Strong and mild peaks are labeled to be better assigned later (Supplementary Table 8). It is clear from this figure that vibration at ~ 1500 cm^{-1} (ii) is much stronger than the rests. This explains its persistence after mixing with carbon conductor when most of other peaks have been covered by noise.

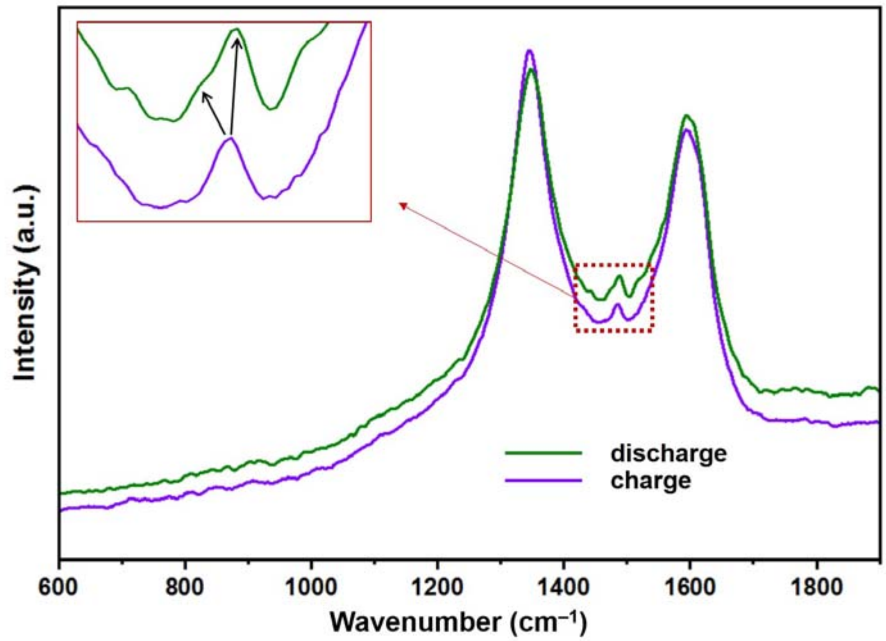

Supplementary Figure 13. Raman shift of charged (purple) and discharged (green) samples. The inset displays the detailed C=O characteristic peaks. It is shown the peak becomes narrower upon charging, indicating the delocalized C=O bond in LFOx became more localized in the charged state. In addition, the peaks shift to higher wavenumber upon charging (dot line to dash-dot line), indicating the C=O bond is becoming stronger.

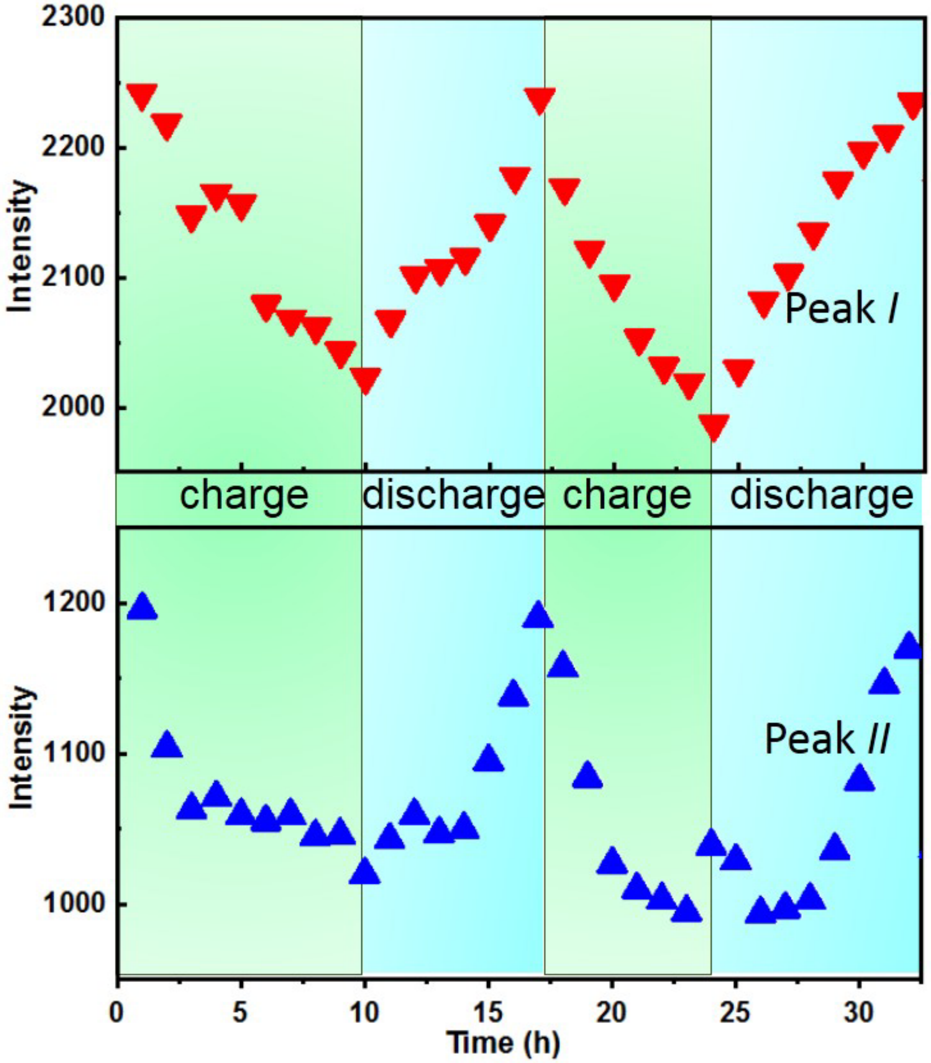

Supplementary Figure 14. Intensity changes of Raman peak *I* and *II* as a function of time in two cycles, corresponding to Fig. 3c,d.

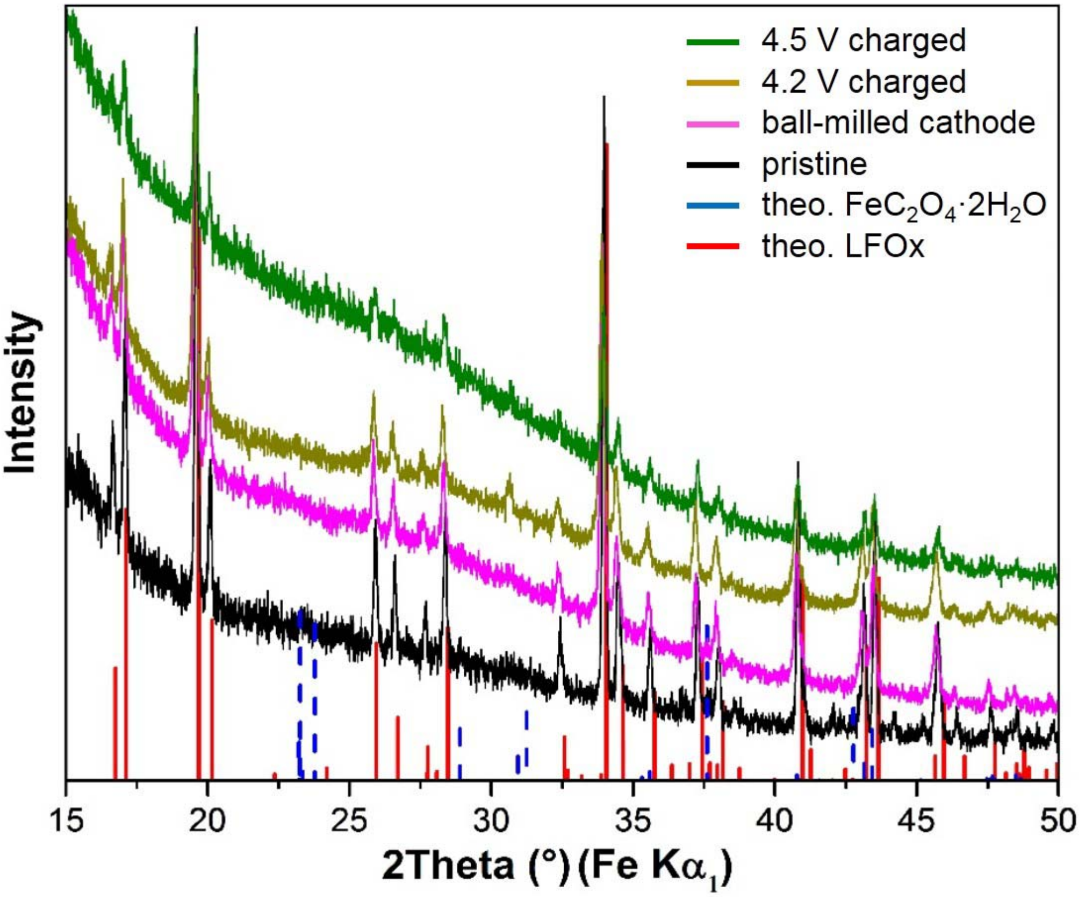

Supplementary Figure 15. XRD patterns of LFOx at different states. Red and blue lines represent standard XRD patterns derived from the crystal structure. Black and magenta curves stand for those on pristine and ball-milled composite samples. Dark yellow and green curves are based on recollected cathode after charging to 4.2 V and 4.5 V. *Ex-situ* samples (dark yellow and green) are rinsed with DMC and dried overnight, and sealed in a capillary. These were operated in the glove box to avoid air contamination. Ball-milled samples showed weaker and broader peaks, demonstrating the size of crystallites were decreased. The capillary samples displayed even less apparent peaks.

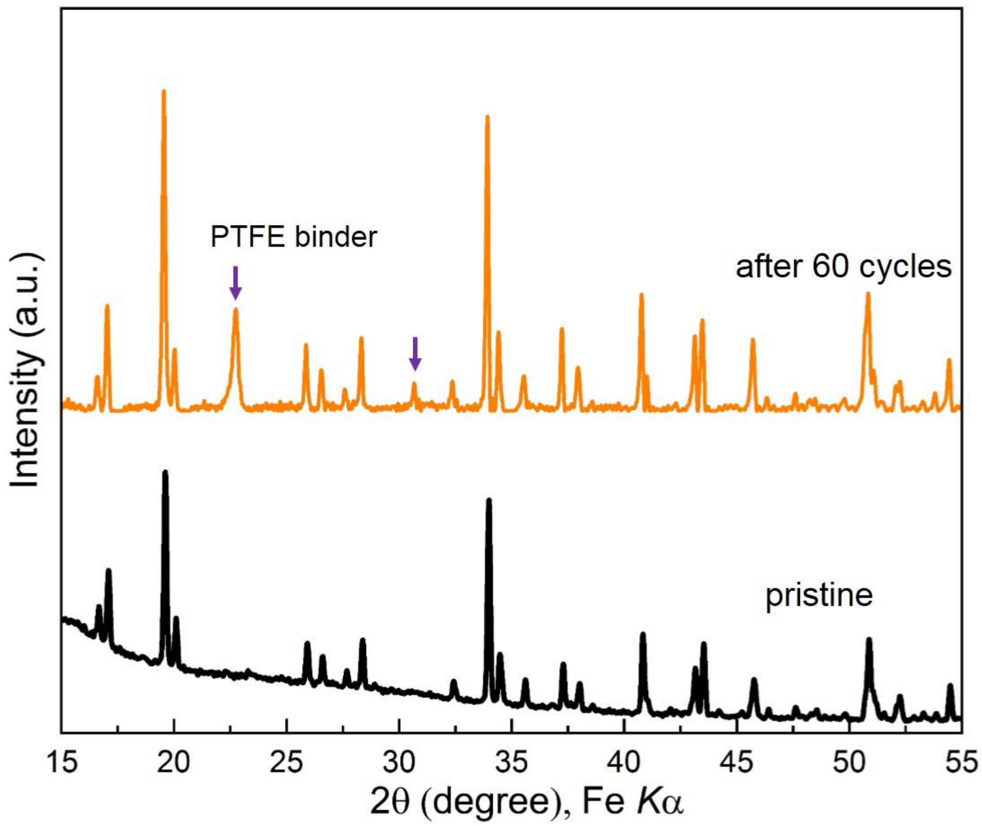

Supplementary Figure 16. XRD patterns of LFOx after 60 (orange) cycles. For comparison, XRD pattern of pristine LFOx is also displayed (bottom black). Recollected samples from coin cells are handled in the glove box and sealed in two plastic papers to avoid contamination from air. It is clear that the profile keeps similar after 60 cycles, indicating the stability of the LFOx during charging and discharging. The arrow-pointed peaks are from PTFE binder.

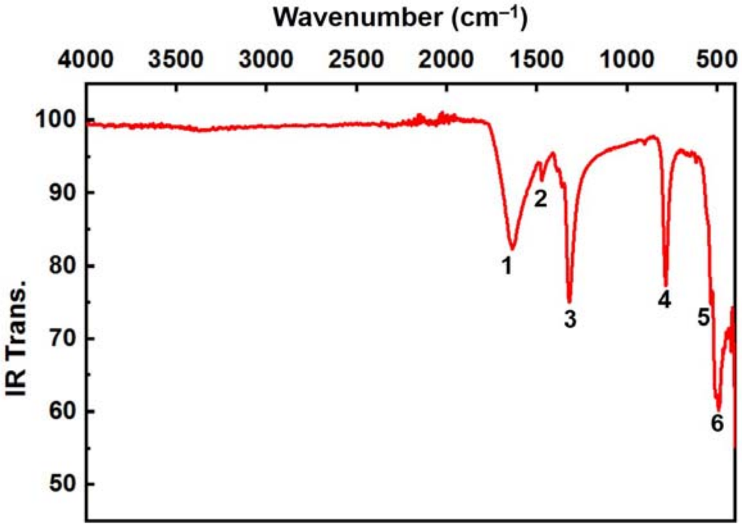

Supplementary Figure 17. IR spectrum of pure LFOx crystallites from 400 – 4000 cm^{-1}. The flat region between 2000 – 4000 cm^{-1} indicates there is no OH group in the sample. Strong peaks are labeled by numbers for easy assignment later (Supplementary Table 8).

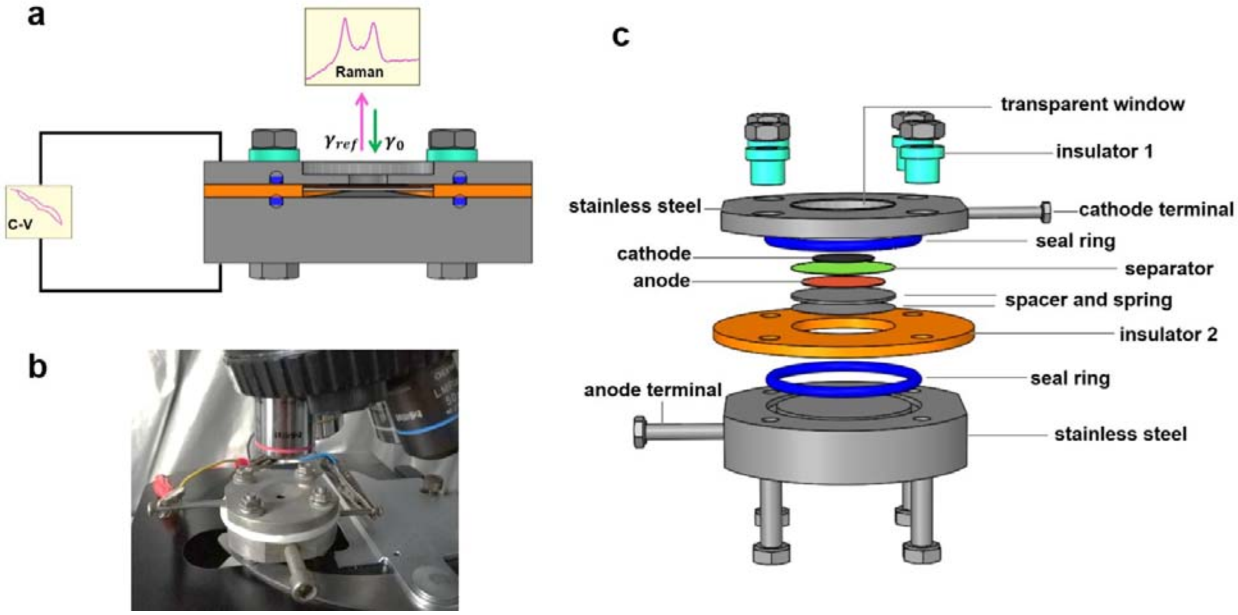

Supplementary Figure 18. Design of *in-situ* Raman spectroscopy. a, Schematic image of facilities combining electrochemical station and Raman Spectrometer. **b**, Photo of a to-be-test *in-situ* cell. **c**, Detailed structure of the self-designed *in-situ* cell.

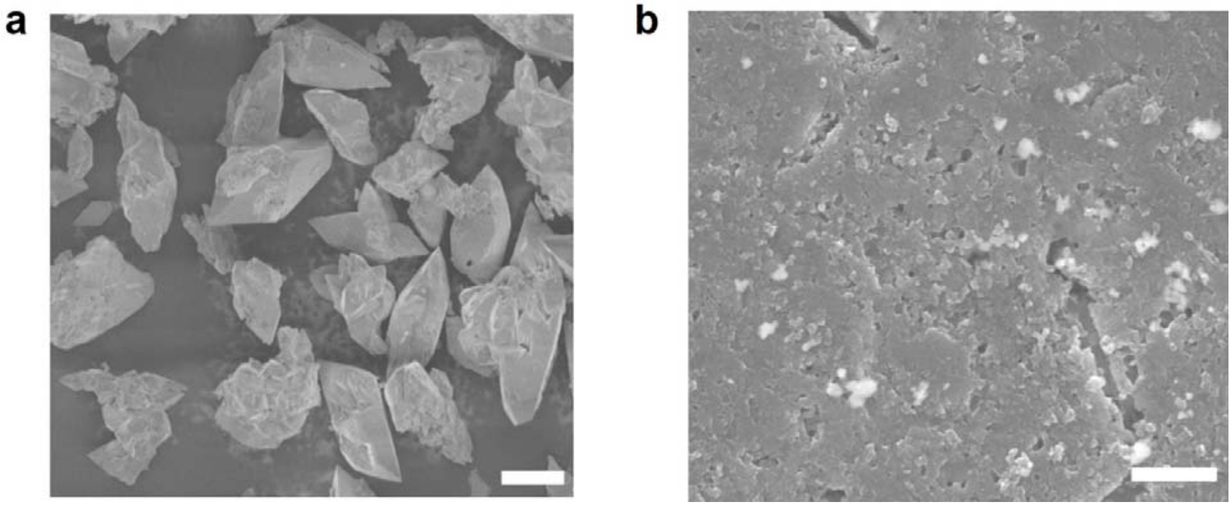

Supplementary Figure 19. SEM image of LFOx at different stage. a, hand-ground sample (scale bar = 20 µm). **b**, Samples after ball milled with carbon conductor (scale bar = 1 µm). The bright particles in **b** are the LFOx in the average size of 100 nm. The dark and black background is the carbon conductor.

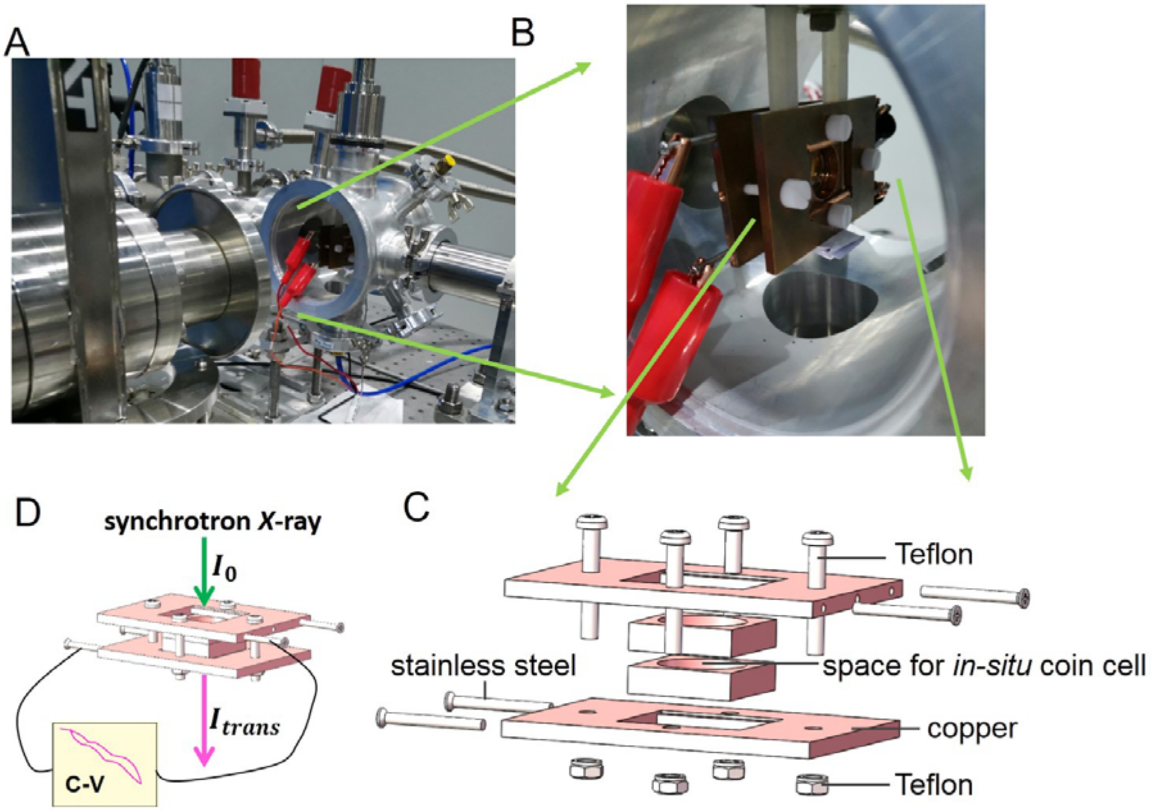

Supplementary Figure 20. Structure of *in-situ* synchrotron X-ray absorption spectroscopy. **a**, a scene photograph of *in situ* cell during testing. **b**, Detailed view of the cell installment. **c**, Detailed structure of the self-designed holder of *in-situ* cell. **d**, Schematic illustration of facilities combining electrochemical station and synchrotron X-ray. B, Photo of a to-be-test *in-situ* cell.

Supplementary Table 1. Contribution of cationic-redox and anionic-redox couples to reversible specific capacity in the representative cathode materials for Li-ion batteries.

Fluorides			Oxides		
Chemical formula	Cationic-redox	Anionic-redox	Chemical formula	Cationic-redox	Anionic-redox
FeF_2 (2 e)[II] [1]	100	0	$LiCoO_2$ [21]	100	0
FeF_3 (1 e) [1]	100	0	$LiMnO_2$ [22]	100	0
FeF_3 (3 e) [1]	100	0	$LiNiO_2$ [23]	100	0
CoF_2 [1]	100	0	$LiMn_2O_4$ [24]	100	0
CuF_2 [1]	100	0	$LiCo_2O_4$ [25]	100	0
$Fe_{0.5}Cu_{0.5}F_2$ [2]	100	0	Li_2MnO_3 [21]	100	0
$FeF_3{\cdot}0.33H_2O$ [3]	100	0	$LiNi_{0.5}Mn_{1.5}O_4$ [21]	100	0
$AgCuF_3$ [4]	100	0	$Li_{1+x}V_{1-x}O_2$ [26]	100	0
BiF_3 [5]	100	0	$Li_{1.2}Ni_{0.13}Mn_{0.54}Co_{0.13}O_2$ [27]	7/12	5/12
Li_3CrF_6 [6]	100	0	$Li_{1.2}Ni_{0.2}Mn_{0.6}O_2$ [28]	100	0
Li_3VF_6 [7]	100	0	$Li_{1.2}Mn_{0.6}Ni_{0.2}O_2$ [28]	100	0
Li_3FeF_6 [8]	100	0	$Li_{1.17}Mn_{0.5}Ni_{0.17}Co_{0.16}O_2$ [28]	100	0
$LiMgFeF_6$ [9]	100	0	$0.5Li_2MnO_3$ 0.5NMC111 [29]	100	0
$LiNiFeF_6$ [10]	100	0	$0.2Li_2MnO_3$ $0.8LiMn_{0.5}Ni_{0.5}O_2$ [29]	100	0
$LiMnFeF_6$ [11]	100	0	$LiNi_{0.8}Co_{0.15}Al_{0.05}O_2$ [30]	100	0
$LiCaFeF_6$ [12]	100	0	Li_3MnO_4 [31]	100	0
Li_2TiF_6 [13]	100	0	$Li_3Mn_{0.7}V_{0.3}O_4$ [32]	100	0
Oxyfluorides			$Li_{1.2}Ti_{0.4}Mn_{0.4}O_2$ [32-b]	80	20
Chemical formula	Cationic-redox	Anionic-redox	Li_2RuO_3 [33]	~5/8	~3/8
$LiFeOF$ [14]	100	0	$Li_2Ru_{0.75}Ti_{0.25}O_3$ [33]	<100	>0
$FeOF$ [15]	100	0	$Li_{1.2}Ni_{0.2}Ru_{0.6}O_2$ [34]	<100	>0
$Fe_{0.9}Co_{0.1}OF$ [16]	100	0	Li_2IrO_3 [34]	2/3	1/3
$MnOF$ [17]	100	0	Li_3IrO_4 [34]	0	100
$BiO_{0.5}F_2$ [18]	100	0	$Li_2Ru_{0.75}Sn_{0.25}O_3$ [34]	62	38
$BiOF$ [18]	100	0	$Li_4Mn_2O_5$ [35]	0	100
Li_2MnO_2F [19]	100	0	V_2O_5 [36]	100	0
Li_2VO_2F[20]	100	0	$V_2O_5{\cdot}2H_2O$ [36]	100	0
Phosphates (P) and related (OH, F, O included)			V_2O_5-550℃ [36]	100	0
Chemical formula	Cationic-redox	Anionic-redox	V_6O_{13} [37]	100	0
$LiFePO_4$ [41]	100	0	$0.6Li_2RuO_3$ $0.4LiCoO_2$ [38]		
$LiCoPO_4$ [41]	100	0	$Li_2Ru_{0.5}Mn_{0.5}O_3$ [38]	80	20
$LiNiPO_4$ [41]	100	0	$1/4Li_2MnO_3$ $3/4LiMn_{3/8}Ni_{3/8}Co_{1/4}O_2$ [29]	279	215
$LiMnPO_4$ [41]	100	0	Li_3CoRuO_5 [38]	100	0
$LiMn_{0.75}Fe_{0.25}PO_4$ [41]	100	0	Li_5FeO_4 [39]	100	0
$LiMn_{0.9}Fe_{0.1}PO_4$ [41]	100	0	Sulphates (S) and related (OH, F, O included)		
$Li_3V_2(PO_4)_3$ [41]	100	0	Chemical formula	Cationic-redox	Anionic-redox
$Li_5V_2(PO_4)_3$ [41]	100	0	$Li_2Fe(SO_4)_2$ [40]	100	0
$FePO_4{\cdot}1.6H_2O$ [41]	100	0	$Li_2Co(SO_4)_2$ [40]	100	0
$FePO_4{\cdot}2H_2O$ [41]	100	0	$Li_2Mn(SO_4)_2$ [40]	100	0

$BiPO_4$ [42]	100	0	$Li_2Ni(SO_4)_2$ [40]		
$Li_3V_2(P_{0.97}B_{0.03}O_4)_3$ [43]	100	0	$Li_2(CoMn)(SO_4)_2$ [40]	100	0
$LiVPO_4F$ [41]	100	0	$Li_2V_2(SO_4)_3$ [40]	100	0
Li_2FePO_4F [41]	100	0	$Li_2Fe_2(SO_4)_3$ [40]	100	0
Li_2MnPO_4F [41]	100	0	$LiCoSO_4OH$ [40]	100	0
Li_2CoPO_4F [41]	100	0	$LiMnSO_4OH$ [40]	100	0
Li_2NiPO_4F [41]	100	0	$LiFeSO_4F$ [40]	100	0
$Li_5V_2(PO_4)_2F_5$ [41]	100	0	$LiCoSO_4F$ [40]	100	0
$Li_5V(PO_4)_2F_2$ (1 e) [41]	100	0	$LiNiSO_4F$ [40]	100	0
$Li_{1.1}Na_{0.4}VPO_{4.8}F_{0.7}$ [44]	100	0	$LiCuSO_4F$ [40]	100	0
$Li_2VO(HPO_4)_2$ [45]	100	0	$KFeSO_4F$ [40]	100	0
$LiVO(H_2PO_4)_2$ [45]	100	0	$Fe_2O(SO_4)_2$ [40]	100	0
$Li_4VO(PO_4)_2$ [41]	100	0	$LiCoOSO_4$ [41]	100	0
Li_2VOPO_4 (2 e) [41]	100	0	$LiNiOSO_4$ [41]	100	0
$LiVOPO_4$ (1 e) [41]	100	0	$Li_2Cu_2O(SO_4)_2$ [40]	100	0
$V_4O_3(PO_4)_3$ [44]	100	0	$Li_2VO(SO_4)_2$ [40]	100	0
$LiVPO_4OH$ [41]	100	0	$LiFeSO_4OH$ [40]	100	0
$LiMnPO_4OH$ [41]	100	0	$Fe_2O(SO_4)_2$ [40]	100	0
$LiVP_2O_7$ [41]	100	0	$NaFe_3(SO_4)_2(OH)_6$ [40]	100	0
$Fe_4(P_2O_7)_3 \cdot H_2O$ [41]	100	0	$NaFe_2(SO_4)_2(OH)_6$ [40]	100	0
$Li_2MnP_2O_7$ [41]	100	0	$Na_2Fe(SO_4)_2 \cdot 4H_2O$ [40]	100	0
$LiFe_{1.5}P_2O_7$ [46]	100	0	$KFe_3(SO_4)_2(OH)_6$ [40]	100	0
$Li_2FeP_2O_7$ [41]	100	0	Borates (B) and related		
$Li_2CoP_2O_7$ [41]	100	0	Chemical formula	Cationic-redox	Anionic-redox
$Li_2VOP_2O_7$ [41]	100	0	$LiMnBO_3$ [41]	100	0
$Li_3Mo_4P_5O_{24}$ [47]	100	0	$LiFeBO_3$ [41]	100	0
$NaMnFe_2(PO_4)_3$ [41]	100	0	$LiCoBO_3$ [41]	100	0
$Li_9V_3(P_2O_7)_3(PO_4)_2$ [48]	100	0	$LiMn_{0.5}Co_{0.5}BO_3$ [50]	100	0
Na3LiFe(P_2O_7) [49]	100	0	$LiMn_{0.5}Fe_{0.4}Mg_{0.1}BO_3$ [51]	100	0
Silicates (Si) and related（OH, F, O included）			$LiMg_{0.1}Co_{0.9}BO_3$ [52]	100	0
Chemical formula	Cationic-redox	Anionic-redox	$Bi_4B_2O_9$ [53]	100	0
Li2MnSiO_4 [41]	100	0	Cu_2FeBO_5 [54]	100	0
Li_2FeSiO_4 [41]	100	0	Fe_3BO_3[54]	100	0
Li_2CoSiO_4 [41]	100	0	$Li_6CuB_4O_{10}$[55]	100	0
Li_2NiSiO_4[59]	100	0	$MgMnB_2O_5$[56]	100	0
$Li_2Mn_{1-x}Fe_xSiO_4$[59]	100	0	$M_3B_2O_6$ (M = Co,Ni,Cu) [57]	100	0
Li_2VOSiO_4[59]	100	0	$Li_7Mn(BO_3)_3$ [58]	100	0
Others					
Chemical formula	Cationic-redox	Anionic-redox	Chemical formula	Cationic-redox	Anionic-redox
$Fe_2(C_2O_4)_3 \cdot 4H_2O$ [60]	100	0	$Li_{0.8}Fe[BP_2O_8] \cdot 3H_2O$ [64]	100	0
$Na_2Fe(C_2O_4)F_2$ [61]	100	0	$(NH_4)_{0.75}Li_{0.25}FePO_4NO_3$[64]	100	0
$Na_2Fe_2(C_2O_4)_3 \cdot 2H_2O$ [62]	100	0	$LiFe(HPO_3)_2$ [64]	100	0
$Li_2[(VO)_2(C_2O_4)(HPO_3)_2]$ [63]	100	0			

[II] the number of transferred electron(s)

Supplementary Table 2. Crystallographic data for $Li_2Fe(C_2O_4)_2$ and atomic sites information

Crystallographic data for $Li_2Fe(C_2O_4)_2$

ICSD No. **1416422** **monoclinic** system, ***P*2₁/*n*** (No. 14), Mr = **245.77** (g/mol)**,** Z = **4**

a = **7.364(3) Å**, *b* = **9.983(3) Å**, *c* = **9.173(4) Å**, *β* = **110.934(14)°** , V = **629.84(13)** Å^3

R (all data) **= 0.0799/0.1337,** Final R *[I > 2σ(I)]* **= 0.0433/0.1078,** *Goof* = **0.883**

Atom	x/a	y/b	z/c	BVS	Atom	x/a	y/b	z/c	BVS
**Fe**	0.10302(10)	0.62061(7)	0.42472(8)	2.06	**O1**	0.1687(5)	0.5301(3)	0.9089(4)	1.676
**Li1**	-0.1033(11)	1.1294(8)	0.7971(9)	1.105	**O2**	0.0773(5)	0.7586(3)	0.5986(4)	1.885
**Li2**	0.3346(12)	0.6401(9)	1.0771(9)	1.025	**O3**	0.0933(5)	0.7550(3)	1.0294(4)	1.907
**C1**	-0.0443(7)	0.7020(5)	0.9249(5)	2.74	**O4**	0.1366(5)	0.4748(3)	0.2644(4)	1.828
**C2**	0.1803(6)	0.9235(5)	0.7976(5)	2.75	**O5**	-0.1094(5)	0.7401(3)	0.2527(4)	1.844
**C3**	-0.0001(7)	0.4259(5)	0.1506(5)	2.77	**O6**	0.1741(5)	0.4674(3)	0.5906(4)	1.987
**C4**	-0.2789(7)	0.6959(4)	0.2081(5)	2.73	**O7**	0.0129(5)	0.9663(3)	0.7558(4)	1.962
					**O8**	0.2823(5)	0.7568(3)	0.3723(4)	1.971

Supplementary Table 3. Anisotropic displacement parameters for LFOx (in $Å^2$)

Atom	U11	U22	U33	U12	U13	U23
**Fe1**	0.0082(3)	0.0093(4)	0.0100(4)	–0.0006(3)	0.0022(2)	0.0013(3)
**O1**	0.0078(17)	0.0141(18)	0.0155(18)	0.0036(13)	–0.0008(13)	–0.0026(14)
**O2**	0.0078(16)	0.0135(17)	0.0090(16)	–0.0004(12)	–0.0008(13)	–0.0038(13)
**O3**	0.0094(17)	0.0108(17)	0.0096(16)	–0.0015(12)	0.0009(13)	–0.0039(13)
**O4**	0.0098(17)	0.0141(17)	0.0087(16)	–0.0002(13)	0.0020(13)	–0.0025(13)
**O5**	0.0069(16)	0.0105(17)	0.0119(17)	–0.0005(12)	–0.0004(13)	0.0009(13)
**O6**	0.0088(16)	0.0086(16)	0.0089(16)	0.0020(12)	0.0011(12)	0.0031(13)
**O7**	0.0085(16)	0.0092(16)	0.0137(17)	0.0001(12)	0.0031(13)	–0.0021(13)
**O8**	0.0068(16)	0.0141(18)	0.0142(17)	–0.0013(12)	0.0012(13)	0.0003(13)
**C1**	0.011(2)	0.007(2)	0.009(2)	–0.0002(17)	0.0032(17)	0.0019(17)
**C2**	0.008(2)	0.006(2)	0.011(2)	–0.0030(17)	0.0029(17)	–0.0013(18)
**C3**	0.008(2)	0.010(2)	0.011(2)	–0.0045(17)	0.0043(17)	0.0028(18)
**C4**	0.010(2)	0.007(2)	0.008(2)	0.0032(17)	0.0005(17)	0.0011(18)
**Li1**	0.011(4)	0.011(4)	0.005(3)	0.002(3)	0.000(3)	0.001(3)
**Li2**	0.015(4)	0.017(4)	0.008(4)	–0.003(3)	–0.005(3)	0.000(3)

Supplementary Table 4. Relation of oxygen classification in Figure 1e and atom sites (Supplementary Table 2) in the unit cell.

Classification	Original atom site<br>Table S1	Percentage
OI	O1	1/8
OII	O3, O7	2/8
OIII	O2, O4, O5, O8	4/8
OIV	O6	1/8

Supplementary Table 5. Room temperature Mössbauer parameters for LFOx at different states corresponding to Fig. 2 b,c and Supplementary Figure 6.

spectrum	Comp.	IS mm s^{-1}	QS mm s^{-1}	LW mm s^{-1}	Abs %	Attribution
4.2 V-Charged (Fig. 2b, up)	Green	1.22	2.43	0.31	88	Fe^{2+}
	Blue	1.19	1.75	0.31	8	Fe^{2+}
	Red	0.46	0.55	0.31	4.0	Fe^{3+}
4.2 V-Charged (Fig. 2b, bottom)	Green	1.22	2.44	0.29	86	Fe^{2+}
	Blue	1.19	1.75	0.29	7	Fe^{2+}
	Red	0.42	0.61	0.29	7	Fe^{3+}
4.5 V-charged (Fig. 2c, up)	Green	1.22	2.40	0.27	57	Fe^{2+}
	Blue	1.11	1.88	0.31	3	Fe^{2+}
	Red	0.38	0.77	0.44	40	Fe^{3+}
4.5 V-charged (Fig. 2c, bottom)	Green	1.23	2.40	0.28	63	Fe^{2+}
	Blue	1.11	1.93	0.25	3	Fe^{2+}
	Red	0.37	0.78	0.47	34	Fe^{3+}
Pristine	Green	1.22	2.39	0.32	92	Fe^{2+}
	Blue	1.19	1.74	0.52	8	Fe^{2+}

Supplementary Table 6. Quantitative analysis of the oxidation state by linear combination fitting of *in-situ* Fe *K*-edge XANES.

	**Sample**	**E (eV)**	**ΔE (eV)**	**oxidation state**	**%Fe^{3+}**	**%Fe^{2+}**
(black dot)	Ref Fe^{2+}	7121.80	0.00	2.000	0	100
(black cross)	Ref Fe^{3+}	7126.80	5.00	3.000	100	0
1 (red)	Original	7123.21	1.41	2.282	28.200	71.800
2 (blue)	C 3.1 V	7122.62	0.82	2.164	16.400	83.600
3	C 3.3 V	7123.03	1.23	2.246	24.600	75.400
4 (purple)	C 3.6 V	7122.62	0.82	2.164	16.400	83.600
5 (olive)	C 3.9 V	7123.60	1.80	2.360	36.000	64.000
6	C 4.1 V	7123.60	1.80	2.360	36.000	64.000
7	C 4.2 V	7122.62	0.82	2.164	16.400	83.600
8	C 4.3 V	7122.80	1.00	2.200	20.000	80.000
9 (magenta)	C 4.5 V	7125.18	3.38	2.676	67.600	32.400
10 (dark yellow)	D 4.2 V	7123.60	1.80	2.360	36.000	64.000
11	D 4.1 V	7123.02	1.22	2.244	24.400	75.600
12 (green)	D 4.0 V	7122.62	0.82	2.164	16.400	83.600
13	D 3.6 V	7123.47	1.67	2.334	33.400	66.600
14 (navy)	D 3.0 V	7123.47	1.67	2.334	33.400	66.600
15	D 2.6 V	7122.62	0.82	2.164	16.400	83.600
16 (orange)	D 2.0 V	7123.60	1.80	2.360	36.000	64.000

C stands for in charging process, and D for discharging process. The data plotted in Figure 2 d-f are signaled by colors in the first column.

Supplementary Table 7. Fourier transforms of the k^2-weighted in situ Fe *K*-edge EXAFS spectra.

	Sample	N	S_0^2	σ_2	e0	delR	R
1 (red)	Original	6	0.716	0.01	6.823	0.06933	2.12103
2 (blue)	C 3.1 V	6	0.719	0.01	5.451	0.04058	2.09227
3	C 3.3 V	6	0.719	0.01	5.489	0.041	2.0927
4 (purple)	C 3.6 V	6	0.721	0.01	5.458	0.04048	2.09218
5 (olive)	C 3.9 V	6	0.721	0.01	5.334	0.04002	2.09172
6	C 4.1 V	6	0.722	0.01	6.345	0.05443	2.10613
7	C 4.2 V	6	0.733	0.01	7.226	0.05687	2.10857
8	C 4.3 V	6	0.730	0.01	7.336	0.05747	2.10917
9 (magenta)	C 4.5 V	6	0.719	0.01	7.059	0.04844	2.10014
10 (dark yellow)	D 4.2 V	6	0.722	0.01	4.908	0.03941	2.09111
11	D 4.1 V	6	0.708	0.01	5.638	0.04204	2.09374
12 (green)	D 4.0 V	6	0.709	0.01	4.95	0.04131	2.09301
13	D 3.6 V	6	0.697	0.01	4.822	0.04298	2.09468
14 (navy)	D 3.0 V	6	0.692	0.01	5.777	0.05077	2.10247
15	D 2.6 V	6	0.672	0.01	6.605	0.05617	2.10787
16 (orange)	D 2.0 V	6	0.688	0.01	5.974	0.05419	2.10589

C stands for in charging process, and D for discharging process. The data plotted in Figure 2 d,f) are signaled by colors in the first column.

Supplementary Table 8. Assignment of IR and Raman spectra of pure LFOx (corresponding to Supplementary Figure 12 & 17).

Peaks number (Supplementary Figure 12 & 17)	IR	Raman	Band assignments
1	1636(strong)		ν_a (C=O)
2	1472		ν_s (C-O) + ν(C-C)
	1386		ν_s (C-O) + δ(O-C=O)
	1359		ν_s (C-O) + δ(O-C=O)
3	1320(strong)		ν s (C-O) + δ(O-C=O)
	904		ν_s (C-O) + δ(O-C=O)
4	784(strong)		δ(O-C=O) + ν(M-O)
5	534(strong)		ν(M-O) + ν(C-C)
6	488 (strong)		Ring deform + δ(O-C=O)
		1741	ν (CO)
i		1643(m)	ν (CO)
		1606	ν (CO)
ii		1486(s)	ν (C=O) stretching
iii		905(m)	ν (C-C) stretching
iv		806	δ(O-C=O) bending
v		605	δ(O-C=O) symmetric bending
vi		508(m)	ν (MO ring)
		435	δ(MO ring)

Supplementary Table 9. Stability test of $Li_{8-x}Fe_4(C_2O_4)_8$, x = 1, 2, 3, 4, 5. When the lattice has been changed from perfect case (scale=1.00) to smaller or bigger case, the calculated total energy in unit of eV has been listed to evaluate the lattice stability.

**scale 0.95**	**scale 0.97**	**scale 1.00**	**scale 1.03**	**scale 1.05**
−389.448	−395.621	**−399.76**	−398.191	−395.22
−394.033	−398.939	**−404.39**	−401.66	−398.219
−397.706	−403.383	**−406.26**	−400.918	−400.015
−404.692	−409.577	**−413.36**	−410.696	−404.152
−410.423	−414.514	**−420.35**	−411.771	−407.864
−416.716	−421.685	**−426.59**	−422.737	−418.369
−421.224	−425.772	**−432.57**	−430.222	−423.765
−428.428	−433.215	**−438.27**	−434.688	−430.538
−432.195	−439.672	**−443.58**	−441.505	−437.955

Supplementary Methods

Mössbauer spectroscopy

In the studied samples, each iron environment is fitted with a doublet with four characteristic parameters, namely below. The fitting results are displayed in Supplementary Table 4.

- Isomer shift (IS): gives mainly the valence of iron (quoted relative to α-Fe standard at room temperature)
- Quadrupole splitting: (QS) gives an idea on the site distortion of electronic charge around iron site
- Line width (LW): narrow when the material is well crystallised (unique site) and broadened when the material is less crystallised, amorphous, has defects etc.
- Relative area (RA): gives the fraction of each type of iron assuming similar Lamb-Mössbauer factors.

Synchrotron X-ray measurements

In the measurements of C and O NEXAFS, *ex-situ* samples were used and samples were exposed to air directly due to the limitation of facilities and techniques. It took about three hours to collect data on each sample, and the sample of the orange line in Fig. 3 f-g was tested at last and therefore exposed in the air for the longest time. As is known, the Fe(II) species tend to be less stable in the air at normal pressure and temperature (NPT) [65], while the oxalate group is more stable at NPT [66]. Therefore, the oxygen NEXAFS is more sensitive to air exposure while carbon NEXAFS is more inert, which possibly results in the discrepancy of orange plots with others.

Mass spectroscopy (MS) measurements

In-situ MS measurements were recorded on an *in-situ* half-cell using the LFOx as cathode to check if the oxalate groups in LFOx was oxidized to CO_2. The *in-situ* MS testing system was sketched in Supplementary Figure 11**a**,**b**. The *in-situ* cell and safety devices were assembled in Ar-filled glove-box. During the experiments, the carrier gas (high-pure Ar) was brought into the electrolyte, so any gas from the electrochemical reaction will be carried out with Ar gas together,

either in the cell headspace or dissolved in the electrolyte. Signals from components of volatile electrolyte and any generated gas can be detected by MS along with that of Ar. Supplementary Figure 11**c**,**d** show standard MS spectra of Ar, CO_2, EC (ethylene carbonate) and DMC (dimethyl carbonate). From the figures, it is indicated that the signal of mass/charge (m/z) at 40 can be used as reference to see if the gas stream is smooth and steady, while the time-resolved signals of m/z at 44 can be used as evidence of CO_2 generation during cell resting and cycling, because the new generated CO_2 would lead to increasing intensities of m/z at 44. Therefore, signals of m/z at 40 and 44 were recorded while the cell was left resting, cycling, charging to 4.5 V and holding at this voltage, and at last discharging to 2.0 V. The resulting signal of m/z at 40 was very stable during the experiment, demonstrating the stability of the testing system. Time resolved signal of m/c = 44 was illustrated in Supplementary Figure 11**e**. It is observed that the intensity of signal m/c = 44 was very steady during the experiment. This gives clear evidence that the battery system was stable, and the LFOx cathode was not oxidized to CO_2 during cycling.

Variant temperature MS spectra were recorded when the battery was holding at 4.5 V-charged state at room temperature, then heat the cell and recorded the temperature and MS patterns. The results were displayed in Supplementary Figure 11**f**. From the figure, it is seen that the signal of m/z = 40 was relatively steady before 60 °C, and became gradually more intensive afterwards.

Calculation

Spin-polarized calculations under the scheme of density functional theory have been carried out for geometry optimizations, total energies, and electronic structures. In these calculations, revised Perdew-Burke-Ernzerhof functional and plane waves with a cut-off energy of 380 eV have been employed, together with the use of ultrasoft pseudopotentials for all elements except hydrogen. The van der Waals interaction has been considered using DFT-D3 scheme [67], as embedded in the VASP code [68]. During geometry optimizations, converges with the force less than 0.02 eV $Å^{-1}$ and energy change less than 10^{-4} eV have been achieved. K-space is sampled by 1×1×1 Monkhorst-Pack k-points.

$Li_2Fe(C_2O_4)_2$ has been simulated by the unit cell, containing four Fe-O units, labelled as $Li_8Fe_4(C_2O_4)_8$, with an optimized triclinic lattice as 7.36 Å × 9.98 Å× 9.17 Å with γ = 110.93°.

The charge and discharge process have been simulated through removing Li-atom one by one from $Li_nFe_4(C_2O_4)_8$, with n varies from 8 to 3. Based on the tests, the lattice keeps well during the Li-migration. The open circuit voltage (OCV) has been derived directly from the energy difference before and after Li intercalation based on the following reaction [69]:

$$Li_nFe_4(C_2O_4)_8 \rightarrow Li_{n-1}Fe_4(C_2O_4)_8 + (Li^+ + e^-) \qquad \text{(Eq. 1)}$$

Then, OCV can be evaluated according to the following formula [70]:

$$OCV = \{(E_{n-1} + E_{Li}) - E_n\}/e \qquad \text{(Eq. 2)}$$

where E_n, E_{n-1} and E_{Li} are the total energies of $Li_nFe_4(C_2O_4)_8$, $Li_{n-1}Fe_4(C_2O_4)_8$ and single Li derived from bulk metallic lithium, respectively. The atomic charges have been analysed using the Bader scheme to partition the system's electrons into atom–atom pairwise components based on the algorithm developed by Henkelman et al [71].

Supplementary References

1. F. X. Wu, G. Yushin. Conversion cathodes for rechargeable lithium and lithium-ion batteries. *Energy Environ. Sci.*, **10**, 433–459 (2017).
2. F. Wang, S. W. Kim, D. H. Seo, K. Kang, L. Wang, D. Su, J. J. Vajo, J. Wang, J. Graetz. Ternary metal fluorides as high-energy cathodes with low cycling hysteresis. *Nat. Com.*, **6**, 6668 (2015).
3. G. H. Chen, X. Z. Zhou, Y. Bai, Y. F. Yuan, Y. Li, M. Z. Chen, L. Ma, G. Q. Tan, J. P. Hu, Z. H. Wang, F. Wu, C. Wu, J. Lu. Enhanced lithium stroage capability of $FeF_3 \cdot 0.33H_2O$ single crystal with active insertion site exposed. *NanoEnergy*, **56**, 884–892 (2019).
4. W. Tong, G. G. Amatucci. Silver copper fluoride: A novel perovskite cathode for lithium batteries. *J. Power Sources*, **362**, 86–91 (2017).
5. M. F. Oszajca, K. V. Kravchyk, M. Walter, F. Krieg, M. I. Bodnarchuk, M. V. Kovalenko. Colloidal BiF_3 nanocrystals: a bottom-up approach to conversion-type Li-ion cathodes. *Nanoscale*, **7**, 16601–16605 (2015).
6. G. Lieser, V. Winkler, H. Geßwein, L. de Biasi, S. Glatthaar, M. J. Hoffmann, H. Ehrenberg, J. R. Binder. Electrochemical characterization of monoclinic and orthorhombic Li_3CrF_6 as positive electrodes in lithium-ion batteries synthesized by a sol-gel process with environmentally benign chemicals. *J. Power Sources*, **294**, 444–451(2015).
7. A. Basaa, E. Gonzalo, A. Kuhn, F. G. Alvarado. Facile synthesis of β-Li_3VF_6: A new electrochemically active lithium insertion material. *J. Power Sources*, **207**, 160–165 (2012).
8. A. Basa1, E. Gonzalo, A. Kuhn, F. G. Alvarado. Reaching the full capacity of the electrode material Li_3FeF_6 by decreasing the particle size to nanoscale. *J. Power Sources*, **197**, 260–266 (2012).
9. G. Lieser, C. Drager, L. de Biasi, S. Indris, H. Geßwein, S. Glatthaar, M. J. Hoffmann, H. Ehrenberg, J. R. Binder. Direct synthesis of trirutile-type $LiMgFeF_6$ and its electrochemical characterization as positive electrode in lithium-ion batteries. *J. Power Sources*, **274**. 1200–1207 (2015).
10. L. de Biasi, G. Lieser; J. Rana, S. Indris, C. Dräger, S. Glatthaar; R. Mönig, H. Ehrenberg, G. Schumacher, J. R. Binder, H. Geßwein. Unravelling the mechanism of lithium insertion into and extraction from trirutile-type $LiNiFeF_6$ cathode material for Li-ion batteries. ***Cryst. Eng. Comm.***, **17**, 6163–6174 (2015).
11. [G. Lieser, L. de Biasi, H. Geßwein, S. Indris, C. Drager, M. Schroeder, S. Glatthaar, H. Ehrenberg, J. R. Bindera. Electrochemical characterization of $LiMnFeF_6$ for use as positive electrode in lithium-ion batteries. *J. Electrochem. Soc.*, **161** (12), A1869–A1876 (2014).
12. L. de Biasi, G. Lieser, C. Drager, S. Indris, J. Rana, G. Schumacher, R. Monig, H. Ehrenberg, J. R. Binder, H. Geßwein. $LiCaFeF_6$: A zero-strain cathode material for use in Li-ion batteries. *J. Power Sources*, **362**, 192–201(2017).
13. I. D. Gocheva, T. Doi, S. Okada, J. Yamaki. Electrochemical properties of trirutile-type Li_2TiF_6 as cathode active material in Li-ion batteries. *Electrochem.*, **8** (5), 471–474 (2010).
14. A. Kitajou, E. Kobayashi, S. Okada. Electrochemical performance of a novel cathode material 'LiFeOF' for Li-ion Batteries. *Electrochem.*, **83** (10), 885–888 (2015).
15. L. P. Wang, T. S. Wang, X. D. Zhang, J. Y. Liang, L. Jiang, Y. X. Yin, Y. G. Guo, C. R. Wang. Iron oxyfluorides as lithium-free cathode materials for solid-state Li metal batteries. *J. Mater. Chem. A*, **5**, 18464 (2017).
16. X. L. Fan, E. Y. Hu , X. Ji, Y. Z. Zhu, F. D. Han, S. Hwang, J. Liu, S. Bak, Z. H. Ma , T. Gao, S. C. Liou, J. M. Bai, X. Q. Yang, Y. F. Mo , K. Xu, D. Su, C. S. Wang. High energy-density and reversibility of iron fluoride cathode enabled via an intercalation extrusion reaction. *Nature Com.*, **9**, 2324 (2018).

17. L. T. Zhang, D. Dambournet, A. Iadecola, D. Batuk, O. J. Borkiewicz, K. M. Wiaderek, E. Salager, M. H. Shao, G. H. Chen, J. M. Tarascon. Origin of the high capacity manganese-based oxyfluoride electrodes for rechargeable batteries. *Chem. Mater.*, **30**, 5362−5372 (2018).

18. M. Bervas, L. C. Klein, and G. G. Amatucci. Reversible conversion reactions with lithium in bismuth oxyfluoride nanocomposites. *J. Electrochem. Soc.*, **153** (1), A159–A170 (2006).

19. R. House, L. Y. Jin, U. Maitra, K. Tsuruta, J. Somerville, D. Forstermann, F. Massel, L. Duda, M. R. Roberts, P. G. Bruce. Lithium manganese oxyfluoride as a new cathode material exhibiting oxygen redox. *Energy Environ. Sci.*, **11**, 926–932 (2018).

20. R. Y. Chen, S. H. Ren, M. Knapp, D. Wang, R. Witter, M. Fichtner, H, Hahn. Disordered lithium-rich oxyfluoride as a stable host for enhanced Li^+ intercalation storage. *Adv. Energy Mater.*, **5**, 1401814 (2015).

21. S. Choi, G. X. Wang. Advanced lithium-ion batteries for practical applications: technology, development, and future perspectives. *Adv. Mater. Technol.*, **3**, 1700376 (2018).

22. A. R. Armstrong, P. G. Bruce. Synthesis of layered $LiMnO_2$ as an electrode for rechargeable lithium batteries. *Nature*, **381**, 499–500 (1996).

23. S. Yamada, M. Fujiwara, M. Kanda. Synthesis and properties of $LiNiO_2$ as cathode material for secondary batteries. *J. Power Sources*, **54**, 209–213 (1995).

24. M. M. Thackeray, P. J. Johnson, L. A.de Picciotto, P. G. Bruce, J. B. Goodenough. Electrochemical extraction of lithium from $LiMn_2O_4$. *Mater. Res. Bull.*, **18**, 461 (1983).

25. S. Choi, A. Manthiram. Synthesis and electrochemical properties of spinel $LiCo_2O_4$ cathodes. Materials for Electrochemical Energy Conversion and Storage, **127**, Chapter 22 (2006).

26. A. R. Armstrong, C. Lyness, P. M. Panchmatia, M. Saiful Islam, P. G. Bruce. The lithium intercalation process in the low-voltage lithium battery anode $Li_{1+x}V_{1-x}O_2$. *Nature Mater.*, **10**, 223–229 (2011).

27. B. Li, C. Li, Z. L. Cao, J. Wang, J. B. Zhao. Improving the electrochemical performance of $Li_{1.2}Mn_{0.52}Co_{0.13}Ni_{0.13}O_2$ by surface nitrogen doping via plasma treatment. *RSC Adv.*, **6**, 31014–31018 (2016).

28. X. Q. Zeng, C. Zhan, J. Lu, K. Amine. Stabilization of a high-capacity and high-power nickel-based cathode for Li-Ion batteries. *Chem.*, **4**, 1–16 (2018).

29. P. K. Nayak, E. M. Erickson, F. Schipper, T. R. Penki, N. Munichandraiah, P. Adelhelm, H. Sclar, F. Amalraj, B. Markovsky, D. Aurbach. Review on challenges and recent advances in the electrochemical performance of high capacity Li- and Mn-Rich cathode materials for Li-Ion batteries. *Adv. Energy Mater.*, **8**, 1702397 (2018).

30. A. Purwanto, C. S. Yudha, U. Ubaidillah, H. Widiyandari, T. Og, H. Haerudin. NCA cathode material: synthesis methods and performance enhancement efforts. *Mater. Res. Express*, **5**, 122001 (2018).

31. J. A. Saint, M. M. Doeff, J. Reed. Synthesis and electrochemistry of Li_3MnO_4: Mn in the +5 oxidation state. *J. Power Sources*, **172**, 189–197 (2007).

32. S. J. Xie, Z. Y. Yu, H. X. Liu, S. Wu. Effects of V doping on the electrochemical performance of Li_3MnO_4 for lithium ion batteries. *Solid State Ionics*, **262**, 102–105 (2014).

33. M. Sathiya G. Rousse, K. Ramesha, C. P. Laisa, H. Vezin, M. T. Sougrati, M-L. Doublet, D. Foix, D. Gonbeau, W. Walker, A. S. Prakash, M. Ben Hassine, L. Dupont, J. M. Tarascon. Reversible anionic redox chemistry in high-capacity layered-oxide electrodes. *Nature Mater.*, **12**, 827–835 (2013).

34. G. Assat, J. M. Tarascon. Fundamental understanding and practical challenges of anionic redox activity in Li-ion batteries. *Nature Energy*, **3**, 373–386 (2018).

35. Z. P. Yao, S. Kim, J. G. He, V. I. Hegde, C. Wolverton. Interplay of cation and anion redox in $Li_4Mn_2O_5$ cathode material and prediction of improved $Li_4(Mn, M)_2O_5$ electrodes for Li-ion batteries. *Sci. Adv.*, **4**, 6754 (2018).

36. C. F. Armer, J. S. Yeoh, X. Li, A. Lowe. Electrospun vanadium-based oxides as electrode materials. *J. Power Sources*, **395**, 414–429 (2018).

37. W. Meng, R. Pigliapochi, P. M. Bayley, O. Pecher, M. W. Gaultois, I. D. Seymour, H. P. Liang, W. Q. Xu, K. M. Wiaderek, K. W. Chapman, C. P. Grey. Unraveling the complex delithiation and lithiation mechanisms of the high capacity cathode material V_6O_{13}. *Chem. Mater.*, **29**, 5513−5524 (2017).

38. L. Croguennec, M. R. Palacin. Recent achievements on inorganic electrode materials for lithium- ion batteries. *J. Am. Chem. Soc.*, **137**, 3140−3156 (2015).

39. M. S. Park, Y. G. Lim, S. M. Hwang, J. H. Kim, J. S. Kim, S. X. Dou, J. Cho, Y. J. Kim. Scalable integration of Li_5FeO_4 towards robust, high performance Lithium ion hybrid capacitors. *Chem. Sus. Chem.*, **7**, 3138–3144 (2014).

40. L. Lander, J. M. Tarascon, A. Yamada. Sulfate-based cathode materials for Li and Na-ion batteries. *Chem. Rec.*, **18**, 1394–1408 (2018).

41. C. Masquelier, L. Croguennec. Polyanionic (phosphates, silicates, sulfates) frameworks as electrode materials for rechargeable Li (or Na) batteries. *Chem. Rev.*, **113**, 6552−6591 (2013).

42. B. N. Hu, X. Y. Wang, Q. L. Wei, H. B. Shu, X. K. Yang, Y. S Bai, H. Wu, Y. F. Song, L. Liu. Bismuth phosphate: a novel cathode material based on conversion reaction for lithium-ion batteries. *J. Alloy. Compd.*, **579**, 18–26 (2013).

43. K. Feng, Y. Cheng, M. R. Wang, H. Z. Zhang, X. F. Li, H. M. Zhang. Synthesis and electrochemical properties of $Li_3V_2(P_{1-X}B_XO_4)_3$/C cathode materials. *J. Mater. Chem. A*, **3**, 19469–19475 (2015).

44. Y.U. Park, D. H. Seo, B. Kim, K. P. Hong, H. Kim, S. Lee, R. A. Shakoor, K. Miyasaka, J. M. Tarascon, K. Kang. Tailoring a fluorophosphate as a novel 4 V cathode for lithium-ion batteries. *Sci. Rep.*, **2**, 704–710 (2012).

45. M. Dubarry, J. Gaubicher, D. Guyomarda, G. Wallez, M. Quarton, C. Baehtz. Uncommon potential hysteresis in the $Li/Li_{2x}VO(H_{2-x}PO_4)_2$ ($0 \leq x \leq 2$) system. *Electrochimica Acta*, **53**, 4564–4572 (2008).

46. C. V. Ramana, A. Ait-Salah, S. Utsunomiya, A. Mauger, F. Gendron, C. M. Julien. Novel lithium iron pyrophosphate ($LiFe_{1.5}P_2O_7$) as a positive electrode for Li-ion batteries. *Chem. Mater.*, **19**, 5319–5324 (2007).

47. B. H. Wen, J. Liu, N. A. Chernova, X. Y. Wang, Y. Janssen, F. Omenya, P. G. Khalifah, M. S. Whittingham. $Li_3Mo_4P_5O_{24}$: a two-electron cathode for lithium-ion batteries with three-dimensional diffusion pathways. *Chem. Mater.*, **28** (7), 2229–2235 (2016).

48. G. F. Gu, D. M. Tang, P. Wu, H. Y. Tian, D. G. Tong. Monodisperse mesoporous $Li_9V_3(P_2O_7)_3(PO_4)_2$ microspheres prepared via a hydrothermal method as cathode material for lithium-ion batteries. *Mater. Lett.*, **92**, 247–251 (2013).

49. H. Kim, I. Park, D. H. Seo, S. Lee, S. W. Kim, W. J. Kwon, Y. U. Park, C. S. Kim, S. Jeon, K. Kang. New iron-based mixed-polyanion cathodes for lithium and sodium rechargeable batteries: combined first principles calculations and experimental study. *J. Am. Chem. Soc.*, **134**, 10369−10372 (2012).

50. V. Ragupathi, J. A. Dinesh, P. Panigrahi, S. Raman, J. Lee, G. S.Nagarajan. $LiMn_{0.5}Co_{0.5}BO_3$ solid solution: towards high performance cathode material for next-generation lithium-ion battery. *Inter. J. Hydro. Energy*, **43**, 4108 –4114 (2018).

51. J. C. Kim, D. H. Seo, G. Ceder. Theoretical capacity achieved in a $LiMn_{0.5}Fe_{0.4}Mg_{0.1}BO_3$ cathode by using topological disorder. *Energy Environ. Sci.*, **8**, 1790–1798 (2015).

52. C. Zor, M. Somera, S. Afyon. $LiMg_{0.1}Co_{0.9}BO_3$ as a positive electrode material for Li-ion batteries. *RSC Adv.*, **8**, 15773–15779 (2018).

53. F. Strauss, G. Rousse, D. Batuk, M. X. Tang, E. Salager, G. Drazic, R. Dominko, J. M. Tarascon. Electrochemical behavior of $Bi_4B_2O_9$ towards lithium-reversible conversion reactions without nanosizing. *Phys. Chem. Chem. Phys.*, **20**, 2330–2338 (2018).

54. C. Martin, A. Maignan, A. Guesdon, F. Laine, O. I. Lebedev. Topochemical approach for transition-metal exchange assisted by copper extrusion: from Cu_2FeBO_5 to Fe_3BO_5. *Inorg. Chem.*, **56**, 2375−2378 (2017).

55. F. Strauss, G. Rousse, D. A. D. Corte, M. B. Hassine, M. Saubanere, M. X. Tang, H. Vezin, M. Courty, R. Dominko, J. M. Tarascon. Electrochemical activity and high ionic conductivity of lithium copper pyroborate $Li_6CuB_4O_{10}$. *Phys. Chem. Chem. Phys.*, **18**, 14960−14969 (2016).

56. A. Debart, B. Revel, L. Dupont, L. Montagne, J.-B. Leriche, M. Touboul, J.-M. Tarascon. Study of the reactivity mechanism of $M_3B_2O_6$ (with M = Co, Ni, and Cu) toward lithium. *Chem. Mater.*, **15**, 3683–3691 (2003).

57. H. F. J. Glass, Z. G. Liu, P. M. Bayley, E. Suard, S.-H. Bo, P. G. Khalifah, C. P. Grey, S. E. Dutton. $Mg_xMn_{2-x}B_2O_5$ pyroborates ($2/3 \leq x \leq 4/3$): high capacity and high rate cathodes for Li-ion batteries. *Chem. Mater.*, **29**, 3118−3125 (2017).

58. S. Afyon, M. Wçrle, R. Nesper. A Lithium-rich compound $Li_7Mn(BO_3)_3$ containing Mn^{2+} in tetrahedral coordination: a cathode candidate for Lithium-ion batteries. *Angew. Chem. Int. Ed.*, **52**, 12541–12544 (2013).

59. H.-N. Girish and G.-Q. Shao. Advances in high-capacity Li_2MSiO_4 (M = Mn, Fe, Co, Ni.) cathode materials for lithium-ion batteries. *RSC Adv.*, **5**, 98666–98686 (2015).

60. H. Ahouari, G. Rousse, J. Rodriguez-Carvajal, M. T. Sougrati, M. Saubanère, M. Courty, N. Recham, J. M. Tarascon. Unraveling the structure of iron(III) oxalate tetrahydrate and its reversible Li insertion capability. *Chem. Mater.*, **27**, 1631−1639 (2015).

61. W. Yao, M.-T. Sougrati, K. Hoang, J. Hui, P. Lightfoot, A. R. Armstrong. $Na_2Fe(C_2O_4)F_2$: a new iron-based polyoxyanion cathode for Li/Na ion batteries. *Chem. Mater.*, **29** (5), 2167−2172 (2017).

62. W. Yao, M.-T. Sougrati, K. Hoang, J. Hui, P. Lightfoot, A. R. Armstrong. Reinvestigation of $Na_2Fe_2(C_2O_4)_3{\cdot}2H_2O$: an iron-based positive electrode for secondary batteries. *Chem. Mater.*, **29**, 9095−9101 (2017).

63. S. Hameed, M. V. Reddy, N. Sarkar, B. V. R. Chowdari, J. J. Vittal. Synthesis and electrochemical investigation of novel phosphite based layered cathodes for Li-ion batteries. *RSC Adv.*, **5**, 60630–60637 (2015).

64. Hooman Yaghoobnejad Asl. New polyanion-based cathode materials for alkali ion batteries. PhD thesis, 2016, Missouri University of Science and Technology.

65. Pilchin, A. N. & Eppelbaum L.V. On the stability of ferrous and ferric ion oxides and its role in rockes and rock-forming minerals stability. *Scientific Israel*, **6**, 3-4, 119−135 (2004).

66. Dollimore, D. The thermal decomposition of oxalates. A review. *Thermochimica Acta*, **117**, 331−363 (1987).

67. Goerigk, L. & Grimme, S. A general database for main group thermochemistry, kinetics, and noncovalent interactions − assessment of common and reparameterized (meta−) GGA density functionals. *J. Chem. Theory Comput.* **6**, 107−126 (2010).

68. Kresse G. & Joubert, D. From ultrasoft pseudopotentials to the projector augmented-wave method *Phys. Rev. B* **59**, 1758−1775 (1999).

69. Kong, Q., Feng, W., Wang, Q., Gan, L. Y. & Sun, C. SiS nanosheets as a promising anode material for Li-ion batteries: a computational study. *Phys. Chem. Chem. Phys.* **19**, 8563−8567 (2017).

70. Fradera, X., Austen, M. A. & Bader, R. F. W. The Lewis model and beyond. *J. Phys. Chem. A***103**, 304–314 (1999).

71. Henkelman, G., Arnaldsson, A. & Jónsson, H. A fast and robust algorithm for Bader decomposition of charge density. *Comput. Mater. Sci.* **36**, 354−360 (2006).
